# Correction: Evolution of Sexes from an Ancestral Mating-Type Specification Pathway

**DOI:** 10.1371/journal.pbio.1002055

**Published:** 2015-01-09

**Authors:** The PLOS Biology Staff

## Notice of Republication

This article was republished on December 18^th^, 2014, to correct a copyright issue with Figure 1A. The authors inadvertently used a copyrighted image, which has now been replaced with a CC-BY image taken by the first author, Sa Geng. Please download this article again to view the correct version.

**Figure 1:**
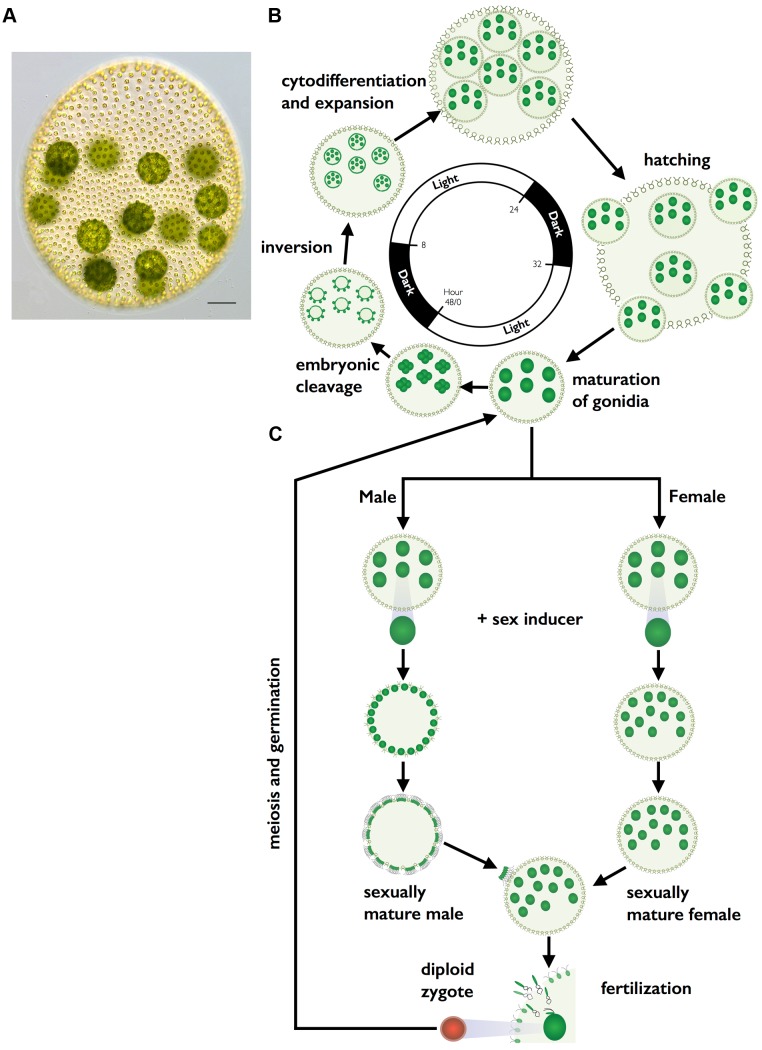
*V. carteri* vegetative and sexual cycles. (A) Color DIC image of vegetative *V. carteri* spheroid with large reproductive cells (gonidia) on the interior and somatic cells on the exterior. Scale bar  =  50 µm. (B) Key stages of the two-day vegetative reproductive cycle are depicted with relative timing indicated by the interior clock diagram showing the 16 h light and 8 h dark phases. Starting from ∼6:00 and going clockwise vegetative gonidia undergo embryonic cleavage followed by inversion to make new juvenile spheroids. Juveniles grow and eventually hatch and mature into the next generation of parental spheroids. The vegetative cycle is identical for males and females. (C) Key stages of the sexual cycle are depicted top to bottom starting with vegetative male or female gonidia that have been exposed to sex-inducer. Sexually induced gonidia undergo modified embryogenesis to produce sexual males with 128 large androgonidia and 128 somatic cells, or sexual females with 32–48 eggs. Subsequent cleavage of androgonidia produces sperm packets that are released and swim to a female whereupon they dissolve into single sperm and enter the female spheroid to fertilize eggs. Diploid zygotes differentiate into environmentally resistant, orange-pigmented, dormant zygospores that when germinated undergo meiosis and produce three polar bodies plus a single haploid vegetative progeny that can reenter the vegetative reproductive cycle.

## Supporting Information

File S1 Republished corrected article.
(PDF)
