# Supplementary figures and images for: Evolution of Sexes from an Ancestral Mating-Type Specification Pathway
Source: PLoS Biol. 2014 Jul 8;12(7):e1001904. doi: 10.1371/journal.pbio.1001904 (PMC4086717; doi:10.1371/journal.pbio.1001904)

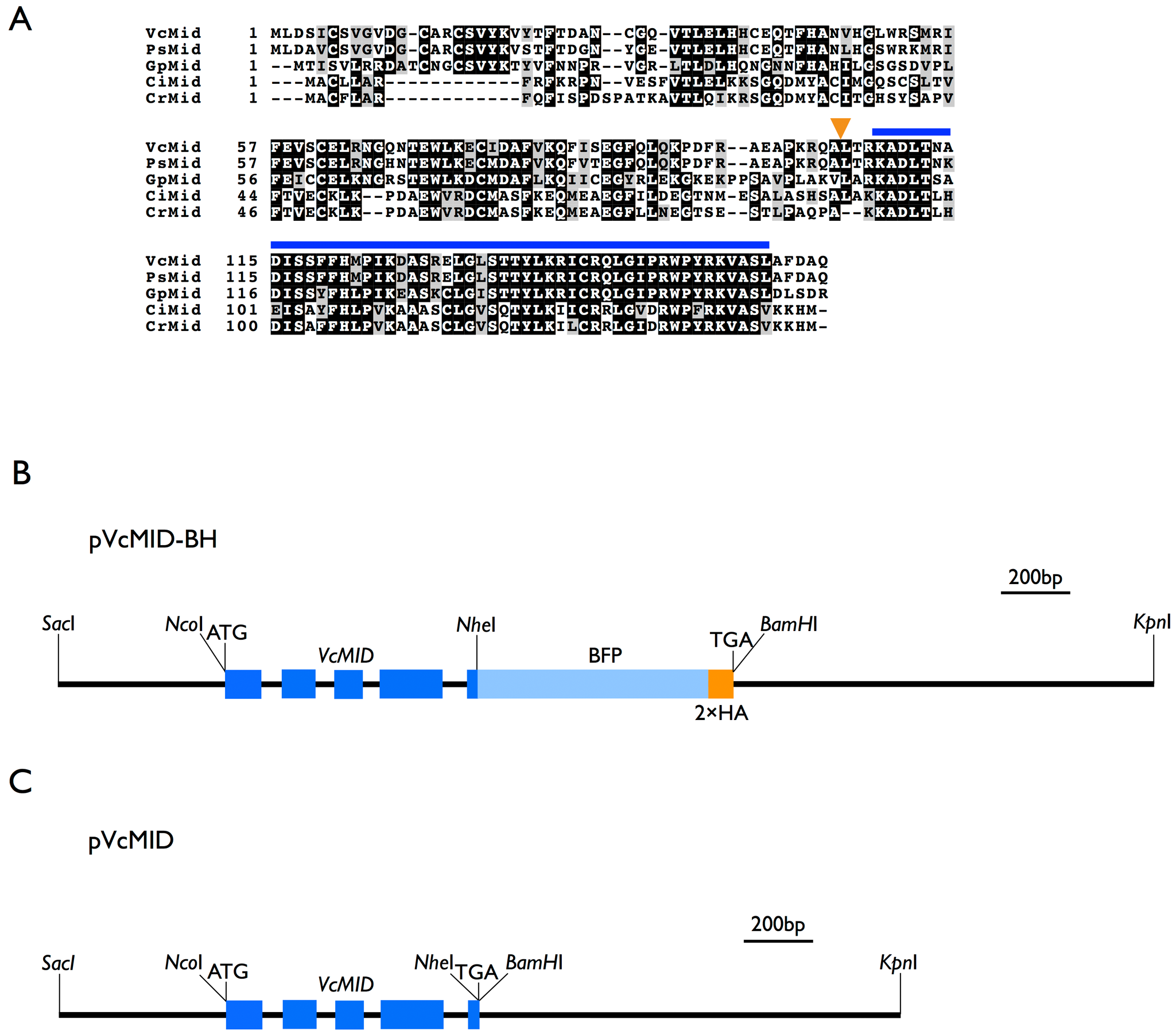

Supplement: Figure S1 — Mid protein alignment and diagrams of pVcMID and pVcMID-BH constructs. (A) Multiple sequence alignment of Mid orthologs from Volvocine algae generated by MUSCLE [74]. The blue line above the sequences demarcates the RWP-RK domain. Species abbreviations are Cr, Chlamydomonas reinhardtii; Ci, Chlamydomonas incerta; Gp, Gonium pectorale; Ps, Pleodorina starrii; Vc, Volvox carteri. Sequences and alignments were described previously [20],[30]. The orange triangle marks the junction point between the N-terminal domain and RWP-RK domain of Mid chimeras described in Figure 5. (B) pVcMID-BH and (C) pVcMID plasmid constructs. Blue filled boxes, exons; black lines, non-coding regions; light blue box, BFP coding sequence; orange box, tandem hemagglutinin (2× HA) epitope tag. Locations of Start (ATG) and termination (TGA) codons as well as relevant restriction enzyme sites are shown. Scale bar is in upper right. (TIF) [file pbio.1001904.s001.tif]

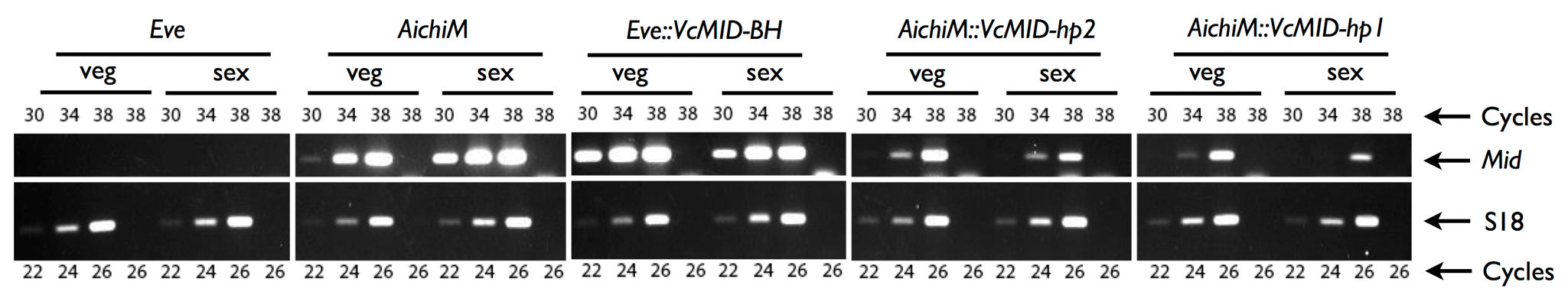

Supplement: Figure S2 — VcMID expression in wild-type and transgenic strains. Gel images from semi-quantitative RT-PCR show VcMID expression levels in indicated strains. RNA from mature vegetative (veg) or mature sexual (sex) spheroids was used for cDNA synthesis and amplification with VcMID primers or with ribosomal protein gene S18 primers as an internal control. Reactions were stopped at the indicated cycle numbers below or above each lane and used for agarose gel electrophoresis followed by ethidium bromide staining and visualization. A negative control reaction without added template was included in each experiment and loaded in the far right lane for each set of reactions. (TIF) [file pbio.1001904.s002.tif]

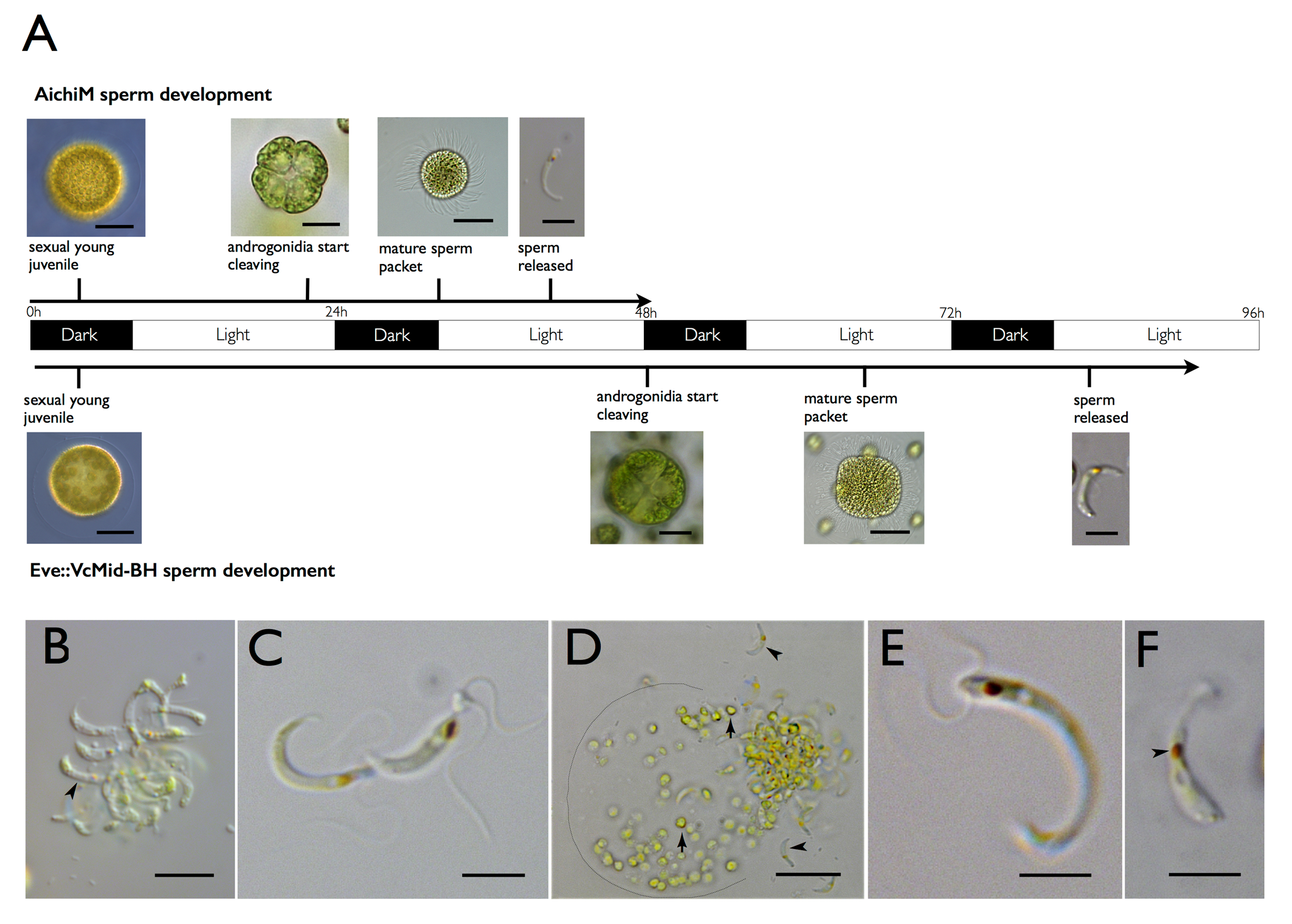

Supplement: Figure S3 — Sperm development in wild-type male strain AichiM and pseudo-male strain Eve::VcMid-BH . (A) The diagram shows a comparison of the developmental chronology for spermatogenesis in wild-type male (AichiM) and pseudo-male (Eve::VcMid-BH) strains. Dark and light boxes in the middle depict successive diurnal cycles (16 h light∶8 h dark). The developmental chronologies start with newly formed, post-embryonic juvenile sexual spheroids (far left). In both strains the timing of development up to this stage is the same. The upper sequence depicts a wild-type male whose androgonidia begin dividing about one day post-embryogenesis. Fully mature sperm packets are formed by the end of the second dark cycle. The sperm packets are released from their parental vesicle and eventually dissociate into individual sperm cells by the middle of the next light cycle. The lower part of the panel shows the same sequence of events for pseudo-male strains whose androgonidia take a full extra day to begin dividing into sperm packets, and whose fully formed sperm packets are delayed in hatching from the parental vesicle. Scale bars for juvenile spheroids = 20 µm, early cleaving androgonidia = 10 µm, mature sperm packets = 25 µm, released sperm = 5 µm. (B) Dissociating sperm packet with wild-type mature sperm from AichiM. Black arrowhead shows a single mature sperm cell. Scale bar = 10 µm. (C) Two individual wild-type sperm cells from AichiM at higher magnification. Scale bar = 5 µm. (D) Dissociating mature sperm packet from Eve::VcMID-BH pseudo-male. The location of the mostly intact vesicle wall surrounding the sperm packet that would normally be dissolved in wild-type males is shown with a dashed line. Black arrowheads indicate cells with relatively normal elongated sperm-like morphology. Black arrows indicate rounded non-sperm-like cells that are only observed in sperm packets of pseudo-males. Scale bar = 20 µm. (E) and (F) Single sperm cells from Eve::VcMID-BH pseudo-males. The sperm cell in [file pbio.1001904.s003.tif]

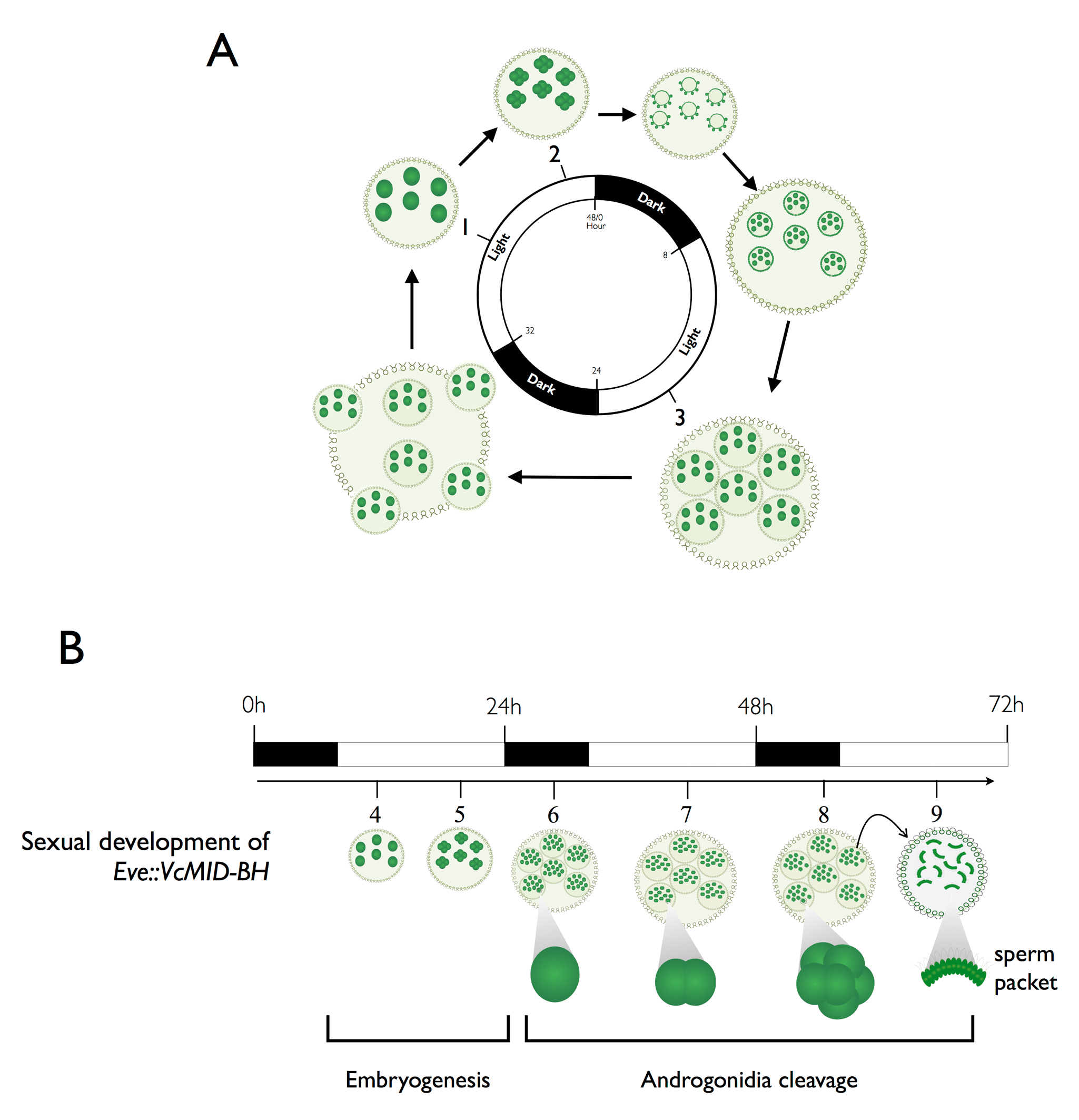

Supplement: Figure S4 — Vegetative and sexual development of Eve::VcMid-BH with sampling time points. (A) Vegetative life cycle diagram of V. carteri. The inner ring shows light (open bar) and dark (closed bar) periods in the 48 hour reproductive cycle. Numbers 1, 2, and 3 show stages at which extracts were prepared for Western blotting in Figure 3A. Images depict key stages starting with mature spheroids at ∼10 o'clock (1) and proceeding clockwise to show cleavage stage (2), pre-inversion stage, early juvenile stage, adult stage (3), and hatching. (B) Diagram showing Eve::VcMid-BH sexual differentiation. Light and dark phases are shown with open and closed bars. Numbers 4–9 show stages at which extracts were prepared for Western blotting in Figure 3A. Diagrammed from left to right are pre-cleavage stage (4), cleavage stage (5), juvenile stage (6), cleaving androgonidia stages (7, 8), and mature stage with sperm packets with a spheroid that has hatched from its parent (9). Images below 6–9 are expanded views of maturation for a single androgonidia showing cleavage into a sperm packet. (TIF) [file pbio.1001904.s004.tif]

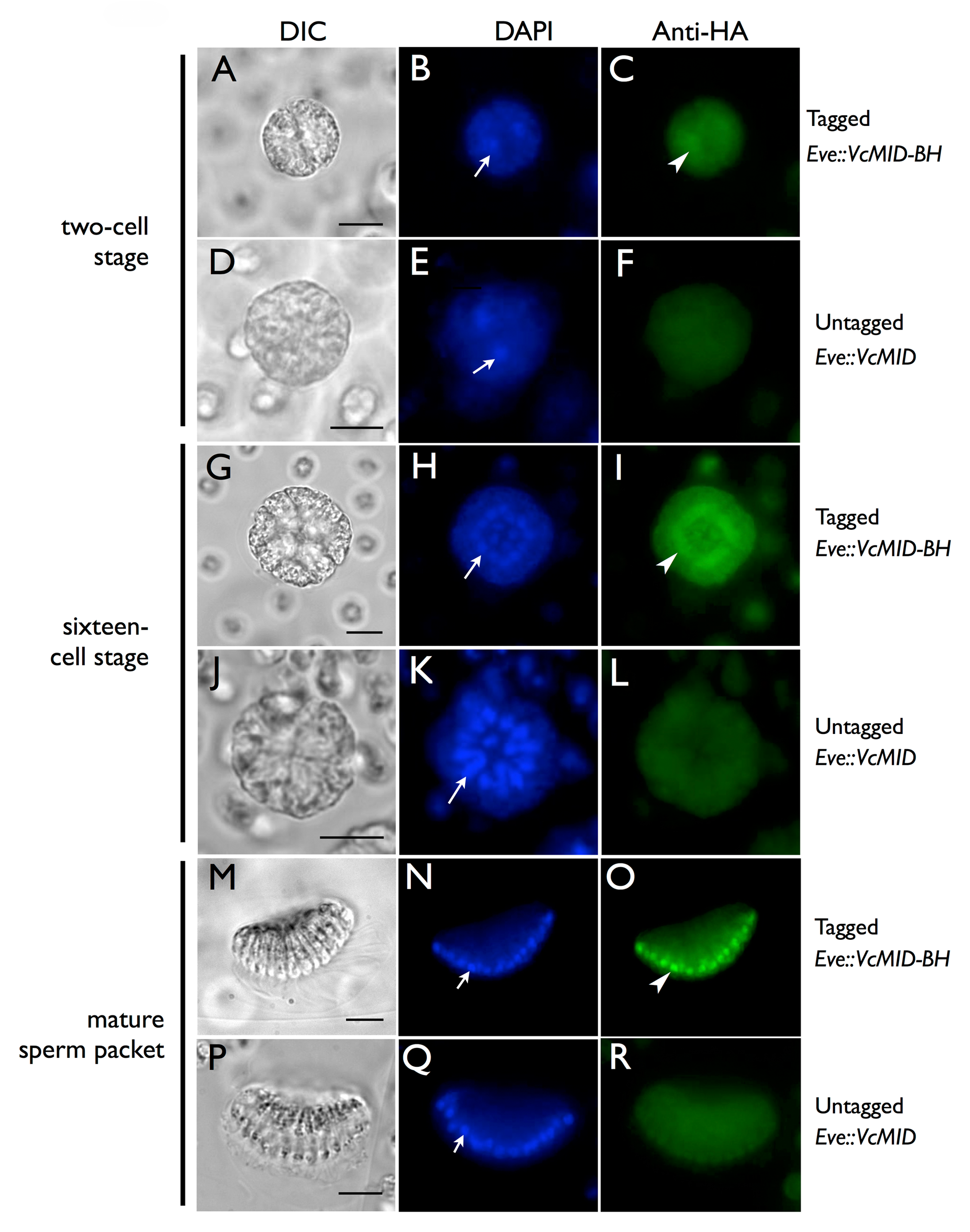

Supplement: Figure S5 — VcMid-BH sub-cellular localization during sperm development. IF images from Figure 2 along with negative control images for each stage. (A–R) Images of two-cell androdonidia (A–F), 16-cell androgonidia (G–L), and mature sperm packets (M–R) from HA-tagged Mid-expressing strain Eve::VcMID-BH (A, B, C, G, H, I, M, N, O) and untagged control transgenic strain Eve::VcMID (D, E, F, J, K, L, P, Q, R). Cells at each stage were imaged by DIC light microscopy (A, D, G, J, M, P), by DAPI fluorescence to visualize DNA in blue (B, E, H, K, N, Q), and by indirect IF to detect the VcMid-BH signal in green (C, F, I, L, O, R). Arrows indicate representative nuclei and arrowheads indicate nuclear staining of VcMid. Scale bars = 10 µm. (TIF) [file pbio.1001904.s005.tif]

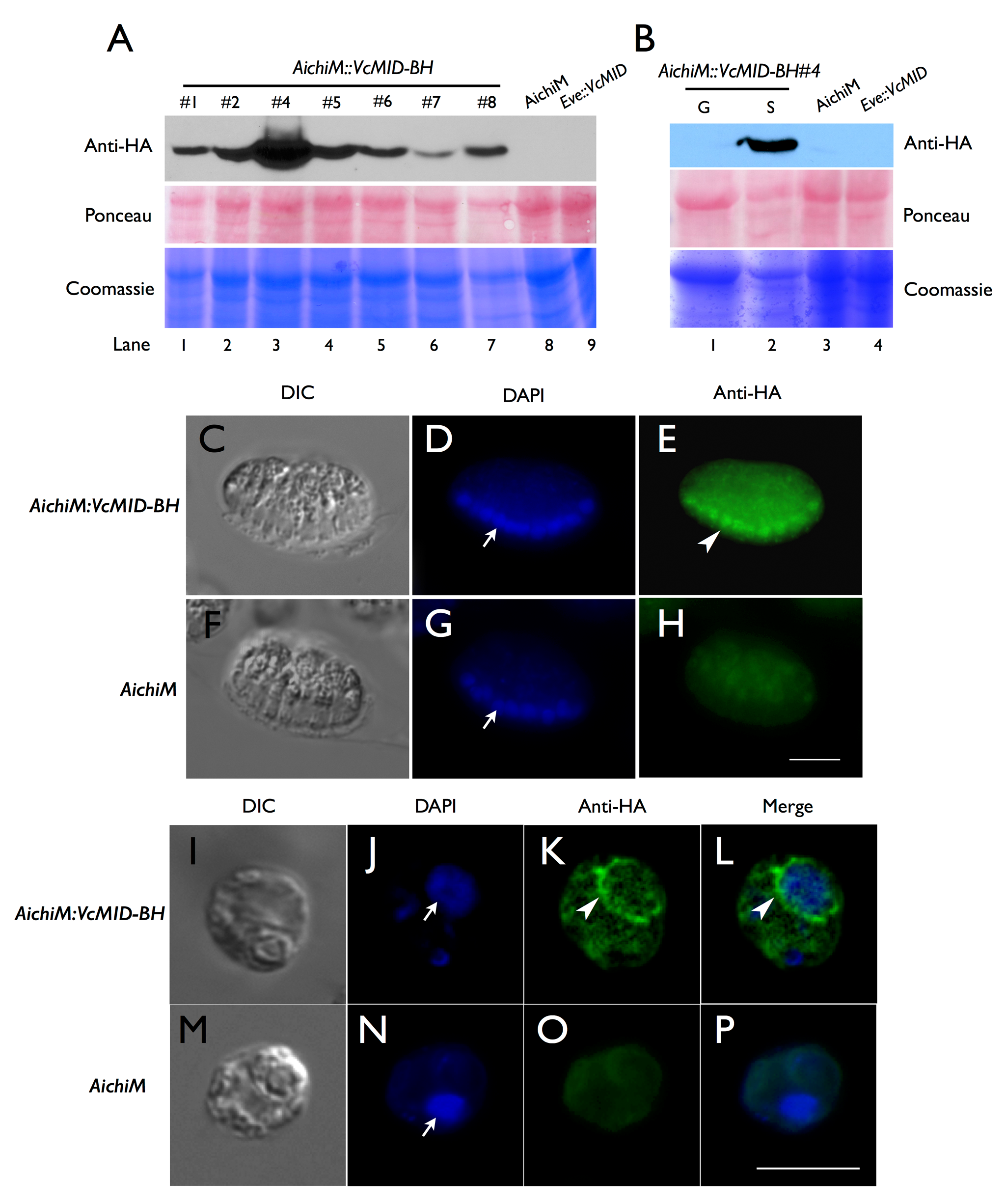

Supplement: Figure S6 — Cell-type restricted expression and sex-regulated nuclear localization of VcMid in wild-type males. (A) Immunoblot of SDS-PAGE fractionated protein extracts from independent AichiM::VcMID-BH transformants (lanes 1–7), wild-type male strain AichiM, and untagged pseudo-male strain Eve::VcMid (lanes 8, 9). The bands in the upper panel are VcMid-BH protein detected with an anti-HA antibody. The bands in the middle panel come from the same blot stained with Ponceau S as a loading control. Lower panel, Coomassie-stained gel with equivalent extract volumes loaded as for Western blot gel. (B) Upper panel, anti-HA immunoblot of SDS-PAGE fractionated protein extracts of purified vegetative gonidia (G) or somatic (S) cells from AichiM::VcMID-BH transformant number 4, wild-type male strain AichiM, and untagged pseudo-male strain Eve::VcMid. The middle and lower panels are the same as in (A). (C–P), DIC (C,F,I,M), or false-colored deconvolved IF images of sperm packet from AichiM::VcMID-BH (C–E) and AichiM (F–H), and of vegetative somatic cell from AichiM::VcMID-BH transformant number 7 (I–L) and vegetative somatic cell from AichiM (M–P). IF samples were stained with DAPI shown in blue (D, G, J, N) or with anti-HA shown in green (E, H, K, L, O, P). The VcMid-BH signal in (K) is mostly outside the nucleus as evident in the merged image (L). Arrows and arrowheads show locations of a representative nucleus from each image. Scale bars = 10 µm in (C–H), and scale bar = 7.5 µm in (I–P). (TIF) [file pbio.1001904.s006.tif]

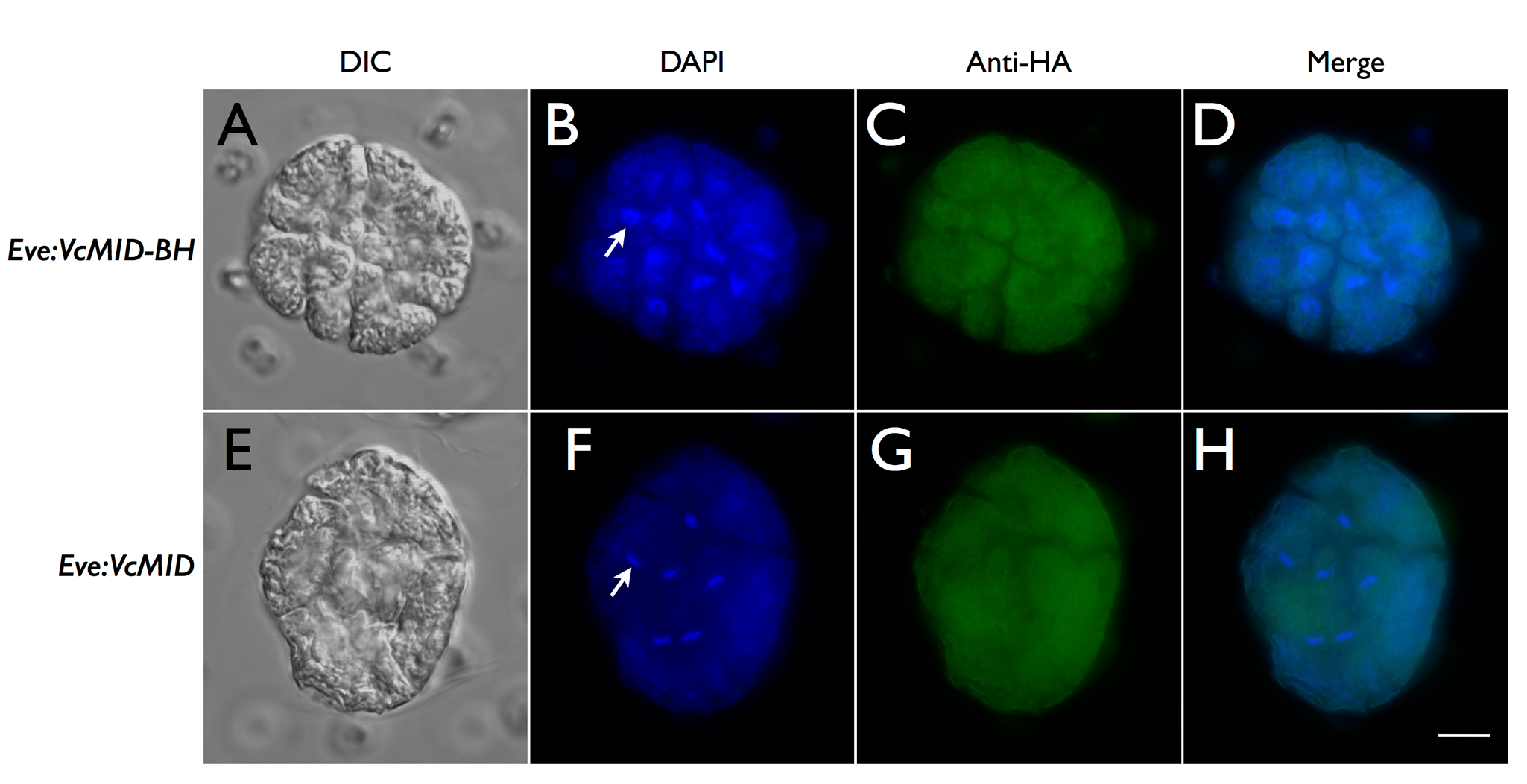

Supplement: Figure S7 — VcMid is not detectable in nuclei of Eve::VcMID-BH embryos undergoing sexual development. DIC (A, E) and fluorescent (B–D, F–H) images of eight or 16 cell stage embryos from Eve::VcMID-BH (A–D) or control Eve::VcMid (E–H) transformants. DAPI staining (B, F) is false colored blue with representative nuclei indicated by arrows. Anti-HA immunostaining (C, G) is false colored green. Merged DAPI and anti-HA images are in (E, H). No nuclear signal for VcMid-BH is detectable above background staining. Scale bar = 10 µm. (TIF) [file pbio.1001904.s007.tif]

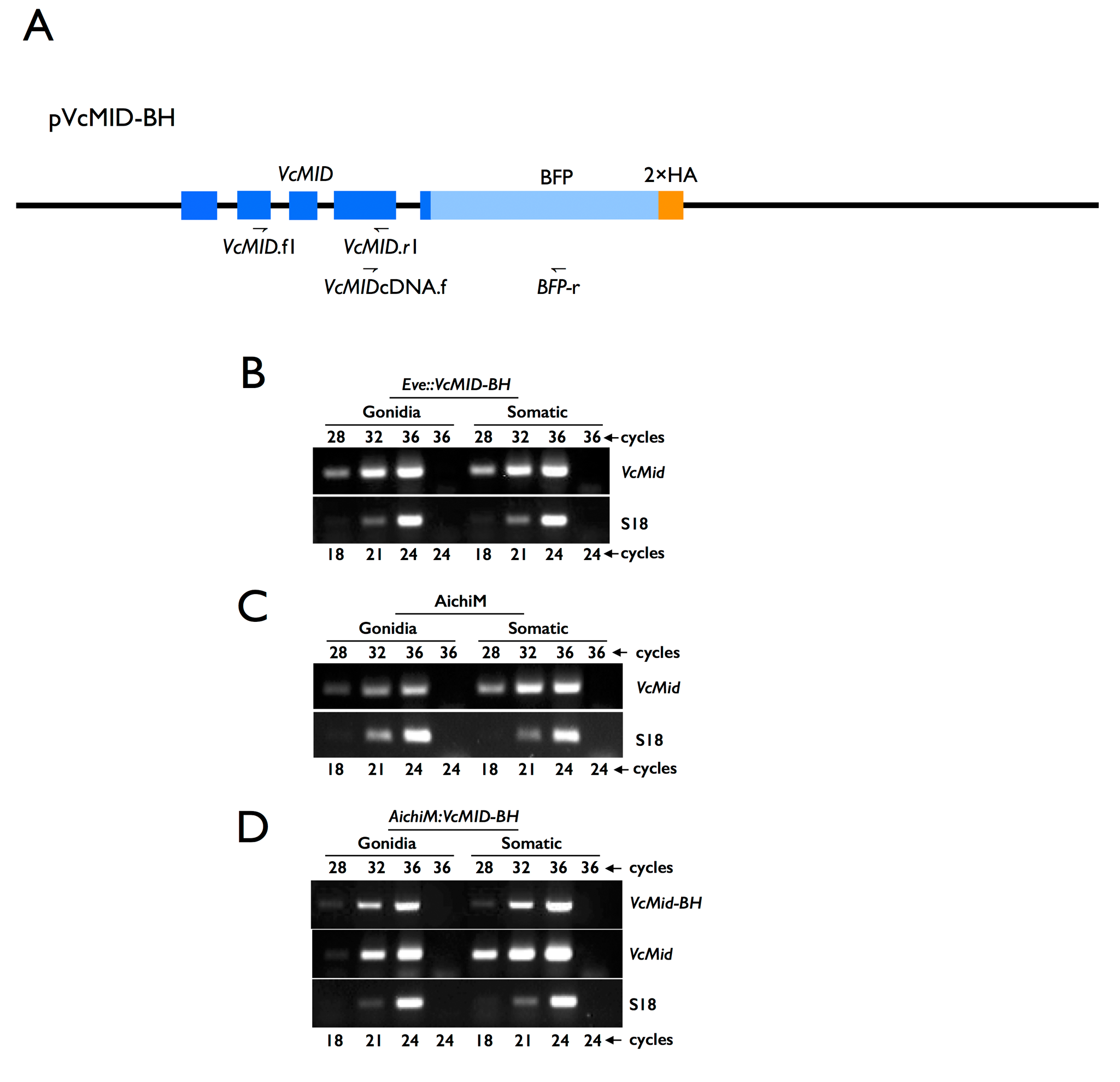

Supplement: Figure S8 — VcMID and VcMID-BH mRNA expression in purified gonidia and somatic cells from V. carteri males. (A) pVcMID-BH diagram showing the location of primers used to amplify the VcMID-BH transgene (VcMIDcDNA.f1 and BFP-r) and the primers that amplify both the endogenous MID gene and VcMID-BH transgene (VcMIDcDNA.f1 and BFP-r). (B–D) Gel images from semi-quantitative RT-PCR show VcMID and/or VcMID-BH expression levels in indicated strains. RNA from gonidia or somatic cells was used for cDNA synthesis and amplification with VcMID primers or with ribosomal protein gene S18 primers as an internal control. Reactions were stopped at the indicated cycle numbers below or above each lane and used for agrose gel electrophoresis followed by ethidium bromide staining and visualization. A negative control reaction without added template was included in each experiment and loaded in the far right lane for each set of reactions. (TIF) [file pbio.1001904.s008.tif]

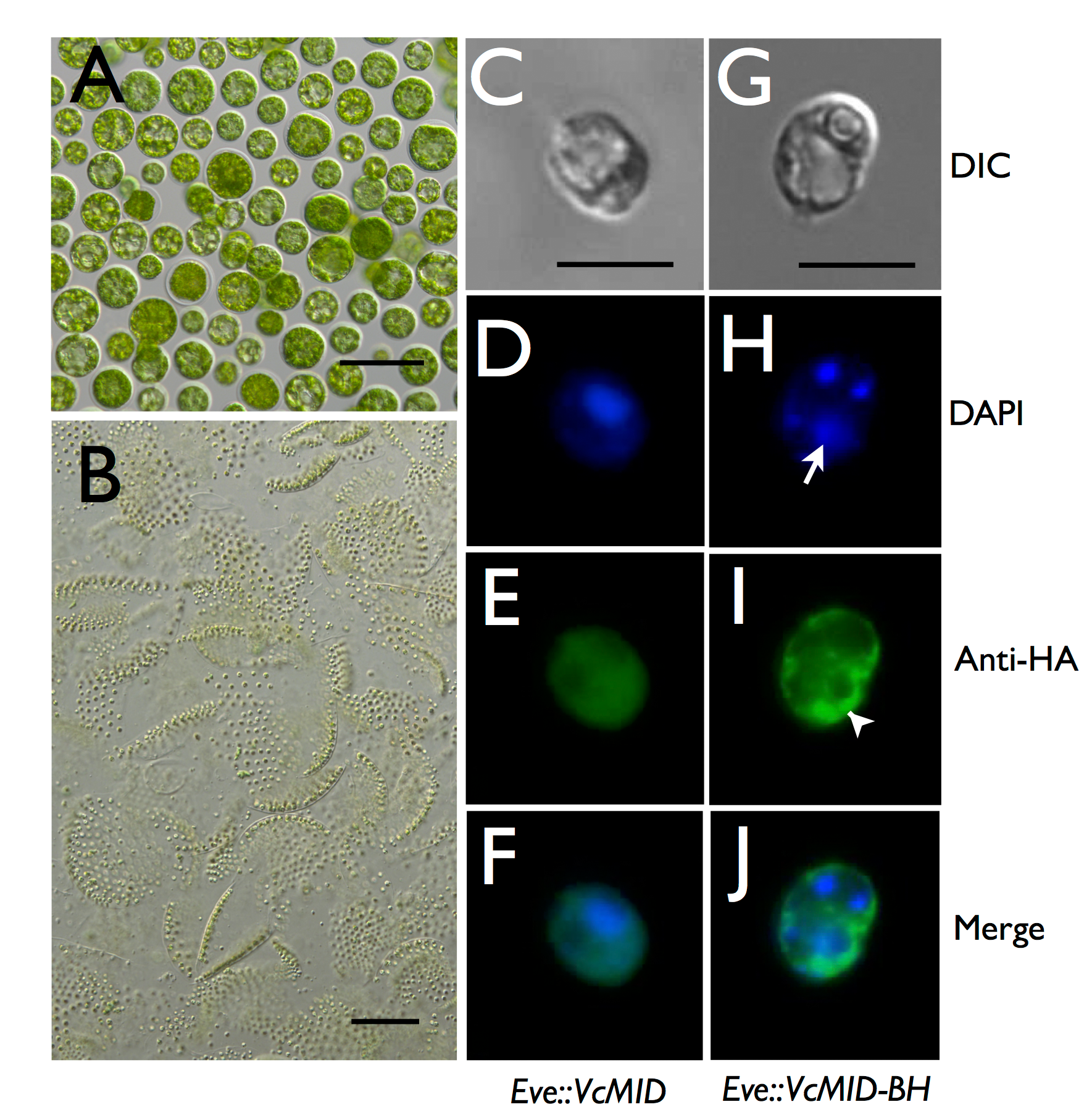

Supplement: Figure S9 — VcMid-BH cell-type expression and subcellular localization in vegetative phase V. carteri . (A) and (B) Microscopic images of purified vegetative gonidia (A) and somatic cells (B) from Eve:: VcMID-BH used for Western blot detection of VcMid. Scale bars, 100 µm in (A) and 200 µm in (B). (C–J) Images of vegetative somatic cells from untagged control strain Eve::VcMID (C–F) or Eve::VcMID-BH (G–J), visualized by DIC light microscopy (C, G), stained with DAPI to visualize DNA in blue (D, H) or subject to IF with anti-HA in green (E, I). (F and J) are merged images of (D, E) and (H, I), respectively. The arrow in (H) shows the nucleus, while the smaller DAPI-stained regions are chloroplast DNA. The arrowhead in (I) shows the cytoplasmic VcMid signal that is excluded from the nucleus of the somatic cell. Scale bars = 7.5 µm. (TIF) [file pbio.1001904.s009.tif]

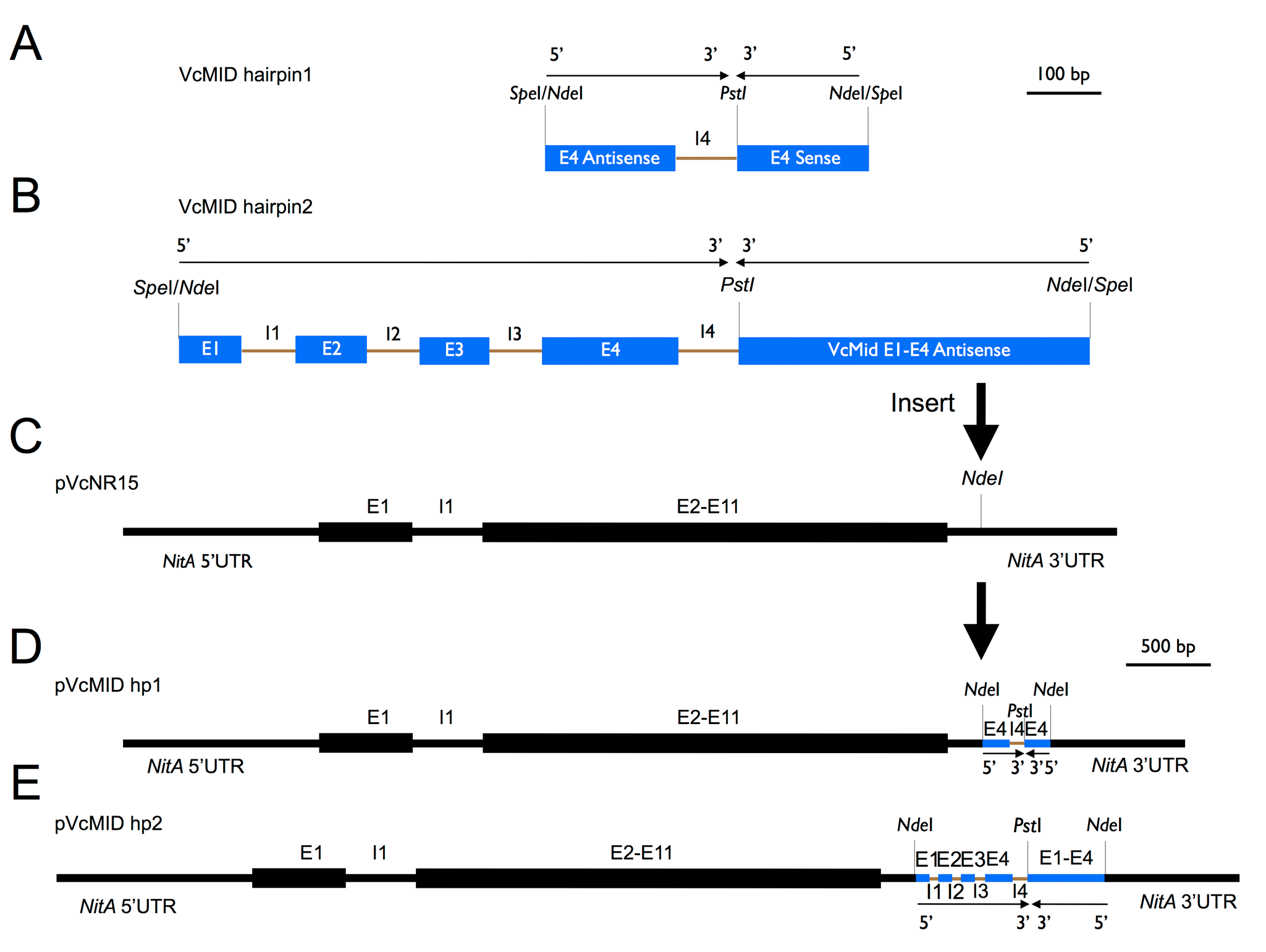

Supplement: Figure S10 — Diagrams of VcMID hairpin constructs. (A) and (B) Sequences used to generate hp1 (A) and hp2 (B) from VcMID genomic and cDNA sequences. Exons E1 through E4 (blue filled boxes) and introns I1 through I4 (orange lines) were amplified and ligated together in the indicated orientations. (C) Diagram of nitA gene in pVcNR15 [71] with exons (E1–E11) indicated by thick black lines and intron 1 or UTR sequences as thin black lines. The Nde I site located in the 3′ UTR region was used as an insertion site for hairpin-forming sequences. (D) and (E) Diagrams of pVcMID-hp1 (D) and pVcMID-hp2 (E) derived from pVcNR15 showing inserted hairpin sequences from (A) and (B) in blue/orange. (TIF) [file pbio.1001904.s010.tif]
