# Supplementary material for: Evolution of Sexes from an Ancestral Mating-Type Specification Pathway
Source: PLoS Biol. 2014 Jul 8;12(7):e1001904. doi: 10.1371/journal.pbio.1001904 (PMC4086717; doi:10.1371/journal.pbio.1001904)
Supplement: Table S1 — Zygote germination and viability in female×male and pseudo-female×male crosses. (DOCX) [file pbio.1001904.s011.docx]

**Table S1. Zygote germination and viability in wild-type female x male and pseudo-female x male crosses**

| **Cross** | **Trial** | **number scored** | **% germination ^1^** | **% germling survival ^2^** | **Total viability ^3^** |
| --- | --- | --- | --- | --- | --- |
| *Eve x AichiM* | 1 | 320 | 81 | 97 | 78 |
| *Eve x AichiM* | 2 | 175 | 74 | 95 | 71 |
| *Eve x AichiM* | 3 | 200 | 64 | 98 | 63 |
| *Eve x AichiM* | **Combined** | **695** | **73 ± 8.4** | **97 ± 1.1** | **70 ± 8.9** |
| *AichiM::MID-hp1 x AichiM* | 1 | 230 | 45 | 49 | 22 |
| *AichiM::MID-hp1 x AichiM* | 2 | 220 | 42 | 53 | 22 |
| *AichiM::MID-hp1 x AichiM* | 3 | 100 | 46 | 48 | 22 |
| *AichiM::MID-hp1 x AichiM* | **Combined** | **550** | **44 ± 1.9** | **50 ± 2.6** | **22 ± 2.1** |

1. Percentage of zygotes that hatched to form a germling spheroid. Combined data from three trials ± standard deviation are shown in bold. 2. Percentage of hatched germling spheroids that survived to produce vegetative offspring. Combined data from three trials ± standard deviation are shown in bold. 3. Total viability calculated as the product of %germination and %germling survival. Combined data from three trials ± aggregated standard deviation are shown in bold.
