# Supplementary material for: Evolution of Sexes from an Ancestral Mating-Type Specification Pathway
Source: PLoS Biol. 2014 Jul 8;12(7):e1001904. doi: 10.1371/journal.pbio.1001904 (PMC4086717; doi:10.1371/journal.pbio.1001904)
Supplement: Table S2 — Gamete differentiation in wild-type and transgenic female Volvox strains expressing Volvox and Chlamydomonas Mid proteins. (DOCX) [file pbio.1001904.s012.docx]

Table S2 Gamete differentiation in wild-type and transgenic Volvox strains

| **Strain** | **Eggs^1^** | **Sperm packets^1^** | **MID transgene expression^2^** |
| --- | --- | --- | --- |
| AichiM | 0% (0/216) | 100% (216/216) | na |
| Eve | 100% (283/283) | 0% (0/283) | na |
| Eve::VcMID-BH | 0% (0/205) | 100% (205/205) | Yes |
| Eve::CrMID-BH#2 | 100% (189/189) | 0% (0/189) | Yes |
| Eve::MID-V_N_C_C_-BH #1 | 100% (186/186) | 0% (0/186) | Yes |
| Eve::MID-C_N_V_C_-BH #6 | 100% (231/231) | 0% (0/231) | Yes |

na, not applicable. 1, number of female spheroids with eggs or sperm packets out of total number spheroids examined. Note that no hermaphrodite phenotypes were observed. 2, detection of HA-tagged Mid protein in strain by Western blot.
