# Supplementary material for: Evolution of Sexes from an Ancestral Mating-Type Specification Pathway
Source: PLoS Biol. 2014 Jul 8;12(7):e1001904. doi: 10.1371/journal.pbio.1001904 (PMC4086717; doi:10.1371/journal.pbio.1001904)
Supplement: Table S3 — Volvox strains used in this study. (DOCX) [file pbio.1001904.s013.docx]

Table S3. *Volvox* strains used in this study.

| Strain Name | Mating Locus | Sexual Phenotype | Transgene | Derived from |
| --- | --- | --- | --- | --- |
| *Eve* (UTEX 1885) | *MTF* | Female | none | na |
| *AichiM* (NIES 398) | *MTM* | Male | none | na |
| *E15* (*nitA^-^*) | *MTF* | Female | none | *Eve* |
| *A18 (nitA^-^)* | *MTM* | Male | none | *AichiM* |
| *Eve::VcMID* | *MTF* | Pseudo-male | *VcMID* | *E15* |
| *Eve::VcMID-BH* | *MTF* | Pseudo-male | *VcMID-BH* | *E15* |
| *Eve::VcMID-hp 1* | *MTF* | Female | *VcMID-hp 1* | *E15* |
| *Eve::VcMID-hp 2* | *MTF* | Female | *VcMID-hp 2* | *E15* |
| *AichiM::VcMID-hp 1* | *MTM* | Pseudo-female | *VcMID-hp 1* | *AichiM* x *Eve::VcMid-hp 1* |
| *AichiM::VcMID-hp 2* | *MTM* | Hermaphrodite | *VcMID-hp 2* | *AichiM* x *Eve::VcMid-hp 2* |
| *Eve::CrMID-BH#2* | *MTF* | Female | *CrMID-BH* | *E15* |
| *Eve::MID-V_N_C_C_-BH#1* | *MTF* | Female | *MID-V_N_C_C_* | *E15* |
| *Eve::MID-C_N_V_C_-BH#6* | *MTF* | Female | *MID-C_N_V_C_* | *E15* |
| *AichiM::VcMID-BH* | *MTM* | Male | *VcMID-BH* | *A18* |

na, not applicable.
