# Supplementary material for: Evolution of Sexes from an Ancestral Mating-Type Specification Pathway
Source: PLoS Biol. 2014 Jul 8;12(7):e1001904. doi: 10.1371/journal.pbio.1001904 (PMC4086717; doi:10.1371/journal.pbio.1001904)
Supplement: Table S4 — Oligonucleotides used in this study. (DOCX) [file pbio.1001904.s014.docx]

Table S4. Oligonucleotides used in this study

| Name | Primer Sequences (5' to 3') |
| --- | --- |
| Plasmid creation | |
| *VcMID*3'UTR.r1 | ACTGAGGTACCGAACAATTACTTTGCAACTGTCGCCATATAAAGC |
| *VcMID*3'UTR.f1 | ACTGAGCTAGCTAAGGATCCATTTGCGATTGCTTGCTCTTCGTACCG |
| *VcMID*GOI.r1 | ACTGAGGATCCTTAGCTAGCTTGGGCATCAAAGGCCAAGCTTGC |
| *VcMID*GOI.f1 | ACTGAGAATTCCTGCAGCCATGGTGGATTCTATTTGTTCCGTTGGCGTC |
| *VcMID*Promoter.r1 | ACTGACCATGGCTGCAGGAATTCCTCAATTCGCAAGATAGGGAAAAGTTAAATGG |
| *VcMID* Promoter.f1 | ACTGAGAGCTCTGGAAGGGAATGGACTCCGGTTCTG |
| *MID*bfp-2XHA F1-2 | GCGGCGGCTACCCGTACGACGTGCCGGACTACTACCCGTACGACGTGCCGGACTACTACCCGTAAGGATCCATTTGCGATTGCTTGCTCT |
| *MID*bfp-2XHA R1 | TTACGGGTAGTAGTCCGGCACGTCGTACGGGTAGTAGTCCGGCACGTCGTACGGGTAGCCGCCGCCGTTCAGCTTATGCCCCAGCTTGCT |
| 3xGly.CrBFP.f1 *Nhe*I | ACTGAGCTAGCGGCGGCGGCATGTCCGAGGAGCTGATCAAGGAGAAC |
| CrBFP.r1 Stp. *BamH*I | ACTGAGGATCCTTATGCCCCAGCTTGCTAGGCAG |
| *CrMID* Promoter.f1 | ACTGAGAGCTCAGGCCTTCTATATATGCGCCGCAAATC |
| *CrMID* Promoter.r1 | ACTGACCATGGCTGCAGGAATTCGCTTGTAGGTATGGAAAGCGCTCTAGG |
| *CrMID* GOI.f1 | ACTGAGAATTCCTGCAGCCATGGCCTGTTTCTTAGCCAGGTTCC |
| *CrMID* GOI.r1 | ACTGAGGATCCCTAGCTAGCCATGTGTTTCTTGACGCTGGCGACC |
| *CrMID*3UTR.f1 | ACTGAGCTAGCTAGGGATCCCGGAGTGCTCGAGCAGCACAC |
| *CrMID*3UTR.r1 | ACTGAGGTACCATACATGGCTGTAAGAAATTAGGGCCCGAGAG |
| CrMidbfp-2XHA F2 | GGCGGCTACCCGTACGACGTGCCGGACTACTACCCGTACGACGTGCCGGACTACTACCCGTAAGGATCCCGGAGTGCTCGAGCAGCACAC |
| CrMidbfp-2XHA R1 | GTAGTCCGGCACGTCGTACGGGTAGTAGTCCGGCACGTCGTACGGGTAGCCGCCGCCGTTCAGCTTATGCCCCAGCTTGCTAG |
| Semi-Quantitative RT-PCR | |
| *VcMID*.f1 | ATTCGAGGTCAGCTGCGAGTTACGA |
| *VcMID*.r1 | TAAGGTACGTCGTGGAAAGCCCCAA |
| S18-1 | GTGGATGGCAAGAACAAGATTATG |
| S18-2 | CTGTAGCGGCCAGTCTTGTG |
| *VcMid*cDNA.f | GACGCGTCACGAGAGTTGGGG |
| *BFP*-r | CGAGCGTCTTCTTCTGCATGAC |
| *VcMID* hairpin creation | |
| *VcMID* *Spe*I*Nde*I E4 f | CTCACTAGTCATATGCGAGCTGAAGCTCCAAAGCGAC |
| *VcMID* *Spe*I*Nde*I E1 f | CTCACTAGTCATATGCCGTTGGCGTCGATGGTTGC |
| *VcMID* *Pst* I E4 r | CCTCTGCAGCTTCCGGTACGGCCAGCGC |
| *VcMID* *Pst* I I4 r | CCTCTGCAGCTGCGAACCAGGGGCAAAAAC |
| Nit- mutant genotyping | |
| *NITA*2.f2 | AGCTCGGACACACCCAAGCTTTTTG |
| *NITA*2.r2 | GGGTGGAGAGAACCGGAGGGATATG |
| *NITA*7.f | ATGTTGCGGAGGTGTGTATGATGT |
| *NITA*7.r | GAGAACTTCCAGTCCGAGCTGTTG |
