# Supplementary material for: Evolution of Sexes from an Ancestral Mating-Type Specification Pathway
Source: PLoS Biol. 2014 Jul 8;12(7):e1001904. doi: 10.1371/journal.pbio.1001904 (PMC4086717; doi:10.1371/journal.pbio.1001904)
Supplement: Text S1 — Supporting methods and references. (DOCX) [file pbio.1001904.s015.docx]

**Text S1. Supporting Methods and References**

**SUPPORTING METHODS**

***Volvox* strains and culture conditions**

*Eve* (*V.c. f. nagariensis* UTEX 1885) and *AichiM* (*V.c. f. nagariensis* NIES 398) were obtained from each of the respective stock centers, http://web.biosci.utexas.edu/utex/ and http://mcc.nies.go.jp/ respectively. Other strains are described in Table S2. All *Volvox* strains were cultured in Standard Volvox Medium (SVM) at 32°C (unless otherwise specified) and synchronized on a 48 hour developmental cycle with a 16hr:8hr light:dark regime[1]. *Eve::VcMID-BH* lines were grown at 28°C for vegetative cultures and 32°C to obtain sexual cultures. Sexual development was induced by adding pre-titered inducer to juveniles 24 hours prior to embryonic cleavage[2].

The nitA- *V. carteri* mutant strains female *E15* and male *A18* that were used for transformations were generated as follows in a manner similar to the procedure of Huskey[3]: 200 cleavage-stage spheroids were grown for two generations in urea-free SVM (UF-SVM) under standard conditions (32°C, 16hr:8hr light:dark), then split into six 350ml flasks of SVM and grown to saturation at 24°C. The flasks were harvested and spheroids washed with 500ml SVM supplemented with 8mM potassium chlorate. The washed spheroids were resuspended in ~380 mL of SVM with 8 mM potassium chlorate and distributed into six 12-well plates for selection of Nit- mutants. After five days of selection chlorate-resistant colonies from positive wells were transferred to fresh SVM supplemented with 8mM potassium chlorate, and to UF-SVM to test for a Nit- phenotype. Nit- mutants were each grown to saturation in 300 mL SVM and re-cultured in 500 mL UF-SVM to identify revertants. Candidates that produced no revertants after 8 days in the above test were further characterized by amplifying portions of the *NitA* locus. *E15* and *A18* were chosen because a region of *NitA* in each strain could not be amplified using the primers *NITA*2.f2 and *NITA*2.r2 or *NITA*7.f and *NITA*7.r (Table S3) where a stable deletion or insertion was presumed to have occurred.

**Plasmid Construction**

The plasmid pVcMID was created as follows: The *VcMID* promoter and 5’ untranslated region (-560bp to -1bp) was amplified from *AichiM* genomic DNA using primers *VcMID* Promoter.f1 and *VcMID* Promoter.r1 that introduced flanking *Sac*I site and *Nco*I restriction sites with the ATG start codon of *VcMID* within the *Nco* I site (ccATGg). The *VcMID* genic region (coding exons and intervening introns,770bp) was amplified using primers *VcMID* GOI.f1 and *VcMID* GOI.r1 that are flanked by *Nco* I and *Nhe* I sites respectively. The *VcMID* 3’ region (1104bp following Stop codon) was amplified with primers *VcMID* 3'UTR.r1 and *VcMID* 3'UTR.f1 that are flanked by *BamH* I and *Kpn* I restriction sites. The above three fragments were digested with the appropriate flanking enzymes, ligated together, and the full length product amplified with primers *VcMID* Promoter.f1 and *VcMID* 3'UTR.r1, and ligated into pGEM-T easy vector (Promega) to obtain pVcMID (Figure S1C ).

The plasmid pVcMID-BH encodes a chimeric fusion of VcMID and C-terminal blue fluorescent protein (BFP) followed by a tandem hemagglutinin (HA) epitope tag. It was constructed from pVcMID as follows: A *Chlamydomonas* codon-biased mTAG-BFP gene[4] was synthesized commercially (IDT, Inc.), and amplified with primers 3xGly.CrBFP.f1 *Nhe*I and CrBFP.r1 Stp. *BamH* I that are flanked by *Nhe* I and *BamH* I restriction sites. The BFP fragment and pVcMID were digested with *Nhe* I and *BamH* I and ligated together to form pVcMID-B. Primer sets *MID*bfp-2XHA R1/ *VcMID* Promoter.f1 and *MID*bfp-2XHA F1-2/ *VcMID*3'UTR.r1 were used to amplify two overlapping fragments from pVcMID-B that were joined into a single fragment containing tandem HA epitopes after the BFP gene by amplification with the primers *VcMID* Promoter.f1 and *VcMID*3’UTR.r1. This fragment was ligated to pGEM-T easy to generate pVcMID-BH. (Figure 1A and Figure S1B).

Plasmid pVcMID-hp1 was created as follows and diagrammed in Figure S10: Primer pairs *VcMID* *Spe*I*Nde*I E4 f /*VcMID* *Pst*I E4 r and *VcMID* *Spe*I*Nde*I E4 f/*VcMID* *Pst*I I4 r were used to amplify *VcMID* exon 4 with flanking *Nde* I and *Pst* I sites as well as *VcMID* exon 4 and intron 4 with flanking *Spe* I*/Nde* I and *Pst* I sites. One of the two fragments was cut with *Spe* I and *Pst*I and one was cut with *Nde I* and *PstI*, then the two fragments were ligated to vector pGEM-T Easy cut with *Spe* I and *NdeI* to generate an inverted repeat. The inverted repeat was excised with *Nde* I and ligated to the *NitA* vector pVcNR15 (Gruber, 1996) digested with *Nde* I. Clones with both orientations of the hairpin in pVcNR15 were mixed in equimolar ratios and used for *Volvox* transformation. pVcMID-hp2 was created in a manner similar to pVcMID-hp1 but using outside flanking primer *VcMID* *Spe*I*Nde*I E1 f instead of *VcMID* *Spe*I/*Nde*I E4 f to generate an inverted repeat hairpin composed of genomic DNA with exons and introns 1-4 in the forward orientation and a cDNA fragment with exons 1-4 in the reverse orientation.

The plasmid pCrMID was created as follows: The *CrMID* promoter and 5’ untranslated region (-674bp to -1bp) was amplified from DNA prepared from wild-type *MT-* *Chlamydomonas* strain CC621 using primers *CrMID* Promoter.f1 and *CrMID* Promoter.r1 that introduced flanking *Sac*I site and *Nco*I restriction sites with the ATG start codon of *CrMID* within the *Nco* I site (ccATGg). The *CrMID* genic region (coding exons and intervening introns,622bp) was amplified using primers *CrMID* GOI.f1 and *CrMID* GOI.r1 that are flanked by *Nco* I and *Nhe* I sites respectively. The *CrMID* 3’ region (1282bp following Stop codon) was amplified with primers *CrMID* 3'UTR.r1 and *CrMID* 3'UTR.f1 that are flanked by *BamH* I and *Kpn* I restriction sites. The above three fragments were digested with the appropriate flanking enzymes, ligated together, and the full length product amplified with primers *CrMID* Promoter.f1 and *CrMID* 3'UTR.r1 and ligated into into pGEM-T Easy (Promega) to obtain pCrMID. pCrMID-BH was created as follows: *Chlamydomonas* codon-biased mTAG-BFP gene[4] was amplified with primers 3xGly.CrBFP.f1 *Nhe*I and CrBFP.r1 Stp. *BamH* I that are flanked by *Nhe* I and *BamH* I restriction sites. The BFP fragment and pCrMID were digested with *Nhe* I and *BamH* I and ligated together to form pCrMID-B. Primer sets CrMidbfp-2XHA R1/ *CrMID* Promoter.f1 and CrMidbfp-2XHA F2/ *CrMID*3'UTR.r1 were used to amplify two overlapping fragments from pCrMID-B that were joined into a single fragment containing tandem HA epitopes after the BFP gene by amplification with the primers *CrMID* Promoter.f1 and *CrMID*3’UTR.r1. This fragment was ligated to pGEM-T easy to generate pCrMID-BH (Figure 5A).

Plasmid pMID-V_N_C_C_-BH was created as follows using Phusion polymerase to amplify all fragments: Primer pair *CrMid*-C-F2/*CrMid*-C-R2 was used to amplify the RWP-RK domain of *CrMID* from *Chlamydomonas* genomic DNA. Primer pair *Vcmid* Promoter.f1/*VcMid*-N-R4 was used to amplify the *VcMID* promoter, 5’ untranslated region and N terminal region from pVcMID-BH. Primer pair *VcMid*BFP-F4/*VcMid*3'UTR.r1 was used to amplify BFP, tandem HA epitopes and the *VcMID* 3’ region from pVcMID-BH. The three fragments were then joined by PCR stitching and amplified using primer pair *Vcmid* Promoter.f1*/ VcMid*3'UTR.r1. The amplified chimeric *MID* gene was ligated to pGEM-T Easy to generate pMID-V_N_C_C_-BH (Figure 5B).

Plasmid pMID-C_N_V_C_-BH was created using a similar stitching strategy as pMID-V_N_C_C_-BH: Primer pair *CrMid*-N-F2/*CrMid*-N-R2 was used to amplify the N-terminal domain of *CrMID* from *Chlamydomonas* genomic DNA. Primer pair *Vcmid* Promoter.f1*/VcMid*pro-R2 was used to amplify the *VcMID* promoter and 5’ untranslated region (-560bp to -1bp) from pVcMID-BH. Primer pair *VcMid*-C-F2/*VcMid*3'UTR.r1 was used to amplify the *VcMID* RWP-RK domain, BFP gene, tandem HA epitopes and 3’ region (1104bp following the Stop codon) from pVcMID-BH. With these three overlapping fragments, primer pair *Vcmid* Promoter.f1*/ VcMid*3'UTR.r1 was used to amplify the fragment containing *VcMID* promoter and 5’ untranslated region, N terminal fragment of *CrMID, VcMID* RWP-RK terminal, BFP gene, tandem HA epitopes and *VcMID* 3’ region. The stitched fragment was ligated to pGEM-T easy to generate pMID-C_N_V_C_-BH (Figure 5C).

**Nuclear transformation of *V. carteri* and generation of transgenic strains**

The *NitA*- *V.carteri* female and male strains *E15* and *A18* were derived from wild-type parental strains *Eve and AichiM* respectively as described above. Cell separation was done just prior to particle bombardment to obtain purified gonidia and/or embryos. Cultures at pre-cleavage or early cleavage stages were collected and transferred to a 40ml Kimble™ Kontes™ Dounce homogenizer. Spheroids were broken with a loose-fitting pestle (A type) using three strokes, then the mixture was transferred to a 50ml Falcon tube and topped up to ~40ml with SVM. 2.8ml Percoll was then added and the mixture spun at 200g for 5min at room temperature. The pelleted gonidia/embryos were washed with ~45ml UF-SVM 3 times and then gently vacuumed onto a sterile membrane filter (Cat#1450055,Whatman) immediately prior to bombardment using a Bio-Rad Helios systems with the following settings: The distance between macrocarrier and stopping screen (Bio-Rad) was set to 5 mm. Rupture disks had a burst pressure of 1100 psi (Bio-Rad, and Inbio Gold,Austalia). The gap between rupture disk and macrocarrier was adjusted to 10mm. The membrane filter with gonidia or embryos was positioned in the bombardment chamber 8cm below the stopping screen. The chamber was partly evacuated to 27 inch Hg prior to bombardment. After each bombardment the membrane filter with embryos/gonidia was transferred to a tube with 20 mL UF-SVM, and incubated under standard growth conditions for 24 hours for females, or 24 hours continuous light for males. Each tube was then divided into a 6-well micro-titer plate containing UF-SVM to undergo selection for *Nit*+ transformants at standard growth conditions for female and at room temperature with continuous light for males. To introduce VcMID hairpin contructs into males we first generated female pVcMID hp1 or pVcMID hp2 transformants (confirmed by genotyping) and then crossed them to wild-type male strain *AichiM*. About half the male progeny from these crosses inherited the RNAi construct and showed a full or partial male-to-female germ cell conversion phenotype as described in the main manuscript. Introducing VcMID-BH into males required direct transformation of male strain *A18* as described above.

**PCR Amplification Conditions**

Genotyping PCR reaction mixtures contained 1-2μL DNA Volvox total DNA prepared as described previously[5] in TE or water, 1μM of each primer, 0.2mM of each dNTP, 1X Ex-Taq buffer (w/2.5mM MgCl2)(Takara), 2% DMSO, and 1-2U of Taq polymerase (Invitrogen, Carlsbad, CA). After 94° for 3 minute, the standard PCR reactions were 36 cycles of 94°C for 30s, 56-60°C for 30s, and 72°C for 2min.

For plasmid construction Phusion PCR reaction mixtures contained 1μM of each primer, 0.2mM of each dNTP, 1X Phusion HF Polymerase Buffer, 5% DMSO, and 1U of Phusion DNA Polymerase (Finnzymes, Woburn, MA). After 30 seconds at 98° the cycling component consisted of 35 cycles of 98°C for 10s, 58-60°C for 30s, and 72°C for 30s. Kentaq LA PCR reaction mixtures contained 1μM of each primer, 0.2mM of each dNTP, 1X Klentaq reaction buffer, 1.3M Betaine, and 0.3μl of Klentaq LA Polymerase (DNA Polymerase Technology). After 2 minutes at 94° the cycling component consisted of 35 cycles of 94°C for 40s, 58°C for 30s, and 68°C for 4min50s.

**SUPPORTING REFERENCES**

1. Kirk DL, Kirk MM (1983) Protein synthetic patterns during the asexual life cycle of Volvox carteri. Dev Biol 96: 493–506.

2. Starr RC, Jaenicke L (1974) Purification and characterization of the hormone initiating sexual morphogenesis in Volvox carteri f. nagariensis Iyengar. Proc Natl Acad Sci USA 71: 1050–1054.

3. Huskey RJ, Semenkovich CF, Griffin BE, Cecil PO, Callahan AM, et al. (1979) Mutants of Volvox carteri affecting nitrogen assimilation. Mol Gen Genet 169: 157–161.

4. Subach OM, Gundorov IS, Yoshimura M, Subach FV, Zhang J, et al. (2008) Conversion of red fluorescent protein into a bright blue probe. Chem Biol 15: 1116–1124. doi:10.1016/j.chembiol.2008.08.006.

5. Jakobiak T, Mages W, Scharf B, Babinger P, Stark K, et al. (2004) The bacterial paromomycin resistance gene, aphH, as a dominant selectable marker in Volvox carteri. Protist 155: 381–393. doi:10.1078/1434461042650343.
