# Supplementary material for: Correction: Evolution of Sexes from an Ancestral Mating-Type Specification Pathway
Source: PLoS Biol. 2015 Jan 9;13(1):e1002055. doi: 10.1371/journal.pbio.1002055 (PMC4289062; doi:10.1371/journal.pbio.1002055)
Supplement: File S1 — Republished corrected article. (PDF) [file pbio.1002055.s001.pdf]

OPEN ACCESS Freely available online

PLOS | BIOLOGY

Evolution of Sexes from an Ancestral Mating-Type Specification Pathway

Sa Geng[1], Peter De Hoff[2¤], James G. Umen[1]*

1 Donald Danforth Plant Science Center, St. Louis, Missouri, United States of America, **2** The Salk Institute for Biological Studies, La Jolla, California, United States of America

Abstract

Male and female sexes have evolved repeatedly in eukaryotes but the origins of dimorphic sexes and their relationship to mating types in unicellular species are not understood. Volvocine algae include isogamous species such as *Chlamydomonas reinhardtii*, with two equal-sized mating types, and oogamous multicellular species such as *Volvox carteri* with sperm-producing males and egg-producing females. Theoretical work predicts genetic linkage of a gamete cell-size regulatory gene(s) to an ancestral mating-type locus as a possible step in the evolution of dimorphic gametes, but this idea has not been tested. Here we show that, contrary to predictions, a single conserved mating locus (*MT*) gene in volvocine algae—*MID*, which encodes a RWP-RK domain transcription factor—evolved from its ancestral role in *C. reinhardtii* as a mating-type specifier, to become a determinant of sperm and egg development in *V. carteri*. Transgenic female *V. carteri* expressing male *MID* produced functional sperm packets during sexual development. Transgenic male *V. carteri* with RNA interference (RNAi)-mediated knockdowns of *VcMID* produced functional eggs, or self-fertile hermaphrodites. Post-transcriptional controls were found to regulate cell-type–limited expression and nuclear localization of VcMid protein that restricted its activity to nuclei of developing male germ cells and sperm. Crosses with sex-reversed strains uncoupled sex determination from sex chromosome identity and revealed gender-specific roles for male and female mating locus genes in sexual development, gamete fitness and reproductive success. Our data show genetic continuity between the mating-type specification and sex determination pathways of volvocine algae, and reveal evidence for gender-specific adaptations in the male and female mating locus haplotypes of *Volvox*. These findings will enable a deeper understanding of how a master regulator of mating-type determination in an ancestral unicellular species was reprogrammed to control sexually dimorphic gamete development in a multicellular descendant.

Citation: Geng S, DeHoff P, Umen JG (2014) Evolution of Sexes from an Ancestral Mating-Type Specification Pathway. PLoS Biol 12(7): e1001904. doi:10.1371/journal.pbio.1001904

Academic Editor: Nick H. Barton, Institute of Science and Technology Austria, Austria

Received March 24, 2014; **Accepted** May 30, 2014; **Published** July 8, 2014

Copyright: © 2014 Geng et al. This is an open-access article distributed under the terms of the Creative Commons Attribution License, which permits unrestricted use, distribution, and reproduction in any medium, provided the original author and source are credited.

Data Availability: The authors confirm that all data underlying the findings are fully available without restriction. All relevant data are within the paper and its Supporting Information files.

Funding: This work was supported by grant NIH R01 GM078376 to J.G.U. The funders had no role in study design, data collection and analysis, decision to publish, or preparation of the manuscript.

Competing Interests: The authors have declared that no competing interests exist.

Abbreviations: BFP, blue fluorescent protein; DIC, differential interference contrast; HA, hemagglutinin; IF, immunofluorescence; MT, mating type locus; RNAi, RNA interference; RT, reverse transcription; SVM, standard volvox medium.

* Email: jumen@danforthcenter.org

¤ Current address: Synthetic Genomics, Inc., La Jolla, California, United States of America

Introduction

In many unicellular and simple multicellular eukaryotes sexual interactions are governed by mating types. Among sexually reproducing organisms mating types are thought to have evolved before gamete size differences and separate sexes evolved [1,2]. Mating types (defined below) control sexual differentiation and specialized roles of cells that function as gametes in a diverse range of taxa including fungi, algae, ciliates, and cellular slime molds [3–6]. In mating-type systems gametes can be isomorphic but can only mate with partners that express a different mating type than their own. Male and female gametes, on the other hand, are a hallmark of multicellular organisms such as metazoans and land plants. Males and females have developmentally specialized gamete types: large immotile eggs that are produced by females or female reproductive organs, and small motile sperm produced by males or male reproductive organs. The groundbreaking theory proposed by Parker and colleagues [7] modeled the evolution of anisogamy (asymmetric-sized gametes) from a starting population of isogametes (i.e., mating types) and identified the evolutionary forces that might cause a mating-type system to evolve into anisogamy (large and small gamete types) or oogamy (eggs and sperm). Additional theories and modifications to the original ideas of Parker and colleagues have been proposed (reviewed in [1,8–10]), but very little attention has been given to the mechanism through which natural selection might act on a mating-type system to drive the transition to anisogamy or oogamy. One model involves the establishment of genetic linkage between a polymorphic locus that affects gamete size and a mating-type locus [11]. However, the genetic basis for the evolution of anisogamy/oogamy has not been determined in any experimental system, and it is not known whether it requires the addition of size control genes or other genes to an ancestral mating locus as the model proposes.

Author Summary

Sexual differentiation in eukaryotes is manifested in two fundamentally different ways. Unicellular species may have mating types where gametes are morphologically identical but can only mate with those expressing a different mating type than their own, while multicellular species such as plants and animals have male and female sexes or separate reproductive structures that produce sperm and eggs. The relationship between mating types and sexes and whether or how an ancestral mating-type system could have evolved into a sexually dimorphic system are unknown. In this study we investigated sex determination in the multicellular green alga *Volvox carteri*, a species with genetic sex determination; we established the relationship of *V. carteri* sexes to the mating types of its unicellular relative, *Chlamydomonas reinhardtii*. Theoretical work has suggested that sexual dimorphism could be acquired by linkage of gamete size-regulatory genes to an ancestral mating-type locus. Instead, we found that a single ancestral mating locus gene, *MID*, evolved from its role in determining mating type in *C. reinhardtii* to determine either spermatogenesis or oogenesis in *V. carteri*. Our findings establish genetic and evolutionary continuity between the mating-type specification and sex determination pathways of unicellular and multicellular volvocine algae, and will enable a greater understanding of how a transcriptional regulator, *MID*, acquired control over a complex developmental pathway.

Volvocine algae are an excellent model for investigating the evolution of sexual dimorphism. They form a monophyletic clade encompassing a progression from unicellular species to multicellular forms with increasing organismal size and cell-type specialization [12,13]. Volvocine algae all have a haploid vegetative reproductive cycle, but under specific conditions can be induced to undergo sexual differentiation and mating to form dormant diploid zygospores. Zygospores undergo meiosis and produce haploid progeny that reenter the vegetative phase [4,14]. *Chlamydomonas* and smaller colonial volvocine genera are isogamous, while larger colonial forms are anisogamous or oogamous as is the case with the genus *Volvox* [15,16]. Some species of *Volvox* and other anisogamous volvocine algae are heterothallic with genetically determined male and female sexes, while others are homothallic with a single clone producing a mixture of all-male and all-female colonies (dioecy), or homothallic with a single clone producing colonies containing both male and female gametes (monoecy) (reviewed in [16]). Previous studies have made use of volvocine algae to evaluate theories relating to the evolution of anisogamy and oogamy [13,17–19], but the genetic basis for sexual dimorphism in this clade is still unclear [4,20,21].

In *C. reinhardtii*, the two genetically determined mating types, *plus* and *minus*, are morphologically similar, but express mating-related genes that allow fusion with a partner of the opposite mating type [14,22]. Gametic differentiation in *C. reinhardtii* is triggered by absence of nitrogen (−N) and is governed by a mating locus (*MT*) whose two haplotypes, *MT*+ and *MT*−, are large, rearranged multigenic regions, which are suppressed for recombination and therefore segregate as Mendelian alleles [23,24]. The *C. reinhardtii* gene *MID* (*CrMID*) is present only in the *MT*− haplotype and encodes a putative RWP-RK family transcription factor whose expression is induced by −N and that governs gametic differentiation [25]. The presence of *MID* activates the *minus* differentiation program and represses the *plus* program, while the absence of *MID* causes activation of the *plus* program and repression of the *minus* program. A second *MT*− gene, *MTD1*, also contributes to *MT*− gametic differentiation but is not essential for it [26]. *MID* is a rapidly evolving gene [27], but orthologs have been found in *MT*− strains or in males of all volvocine algae examined to date including *VcMID* in *V. carteri* (Figure S1A) [20,21,27–30]. However, the role of *MID* in sex determination has not been investigated outside of *Chlamydomonas*.

Volvox carteri f. nagariensis (hereafter *V. carteri*) is a spheroidal multicellular alga whose vegetative form is identical for males and females (Figure 1A). Each vegetative spheroid contains ~2,000 sterile flagellated somatic cells on the periphery that provide motility, while inside the spheroid are ~16 large immotile reproductive cells called gonidia. All of the cells are embedded within a clear extracellular matrix that comprises most of the spheroid volume. The two-day vegetative reproductive cycle begins with mature gonidia undergoing embryogenesis to form new miniature juvenile spheroids. During embryogenesis a programmed series of symmetric and asymmetric cleavage divisions occurs to produce a hollow ball of 2,000 small cells with 12–16 large cells on the anterior surface. The process of inversion then turns the embryo inside out so that the large cells end up on the interior of the spheroid where they will differentiate into new gonidia, and the small cells end up oriented with their basal bodies facing outward, and will begin to grow flagella as they undergo somatic differentiation. Over the next 1.5 days the juveniles grow, mature into adults, hatch, and begin the cycle again (reviewed in (Figure 1B) [31,32]). Unlike *C. reinhardtii* that uses a nutrient trigger for gametogenesis, sexual differentiation in *V. carteri* is triggered by a diffusible glycoprotein hormone called sex-inducer that is active on both sexes [33–35]. In response to sex-inducer, gonidia from vegetative females and males undergo modified embryogenesis programs to produce sexual spheroids (Figure 1C) [36,37]. Sexually induced female spheroids have ~2,000 somatic cells similar to vegetative females, but inside contain 32–48 large egg cells that are formed during embryogenesis through altered timing of asymmetric cell divisions. Sexually induced male spheroids develop with 128 somatic cells and 128 large cells called androgonidia that are also produced through modification of asymmetric embryonic division patterning. The day after male sexual embryogenesis each androgonidial cell undergoes additional cleavage divisions to form a packet of 64 or 128 sperm cells. Sperm packets hatch and swim together to a sexual female where they break apart into individual sperm that enter the female through a fertilization pore. Sperm swim within the female until they find an egg and then fuse with it to form a diploid zygospore. Upon germination a single vegetative meiotic progeny is formed while the remaining three meiotic products are discarded as polar bodies (Figure 1C) [38].

Sexual differentiation in *V. carteri* is controlled by a dimorphic sex-determining locus (*MT*) with haplotypes designated *MTM* (male) and *MTF* (female). *V. carteri MT* occupies an equivalent chromosomal position to *C. reinhardtii MT* based on flanking syntenic gene content, but is at least 5-fold larger. Compared with *C. reinhardtii MT V. carteri MT* contains more sequence rearrangements between haplotypes, more repeat sequences, and has gametolog pairs (genes with an allele in both *MT* haplotypes) that are far more differentiated from each other [20,24]. *V. carteri MTF* and *MTM* haplotypes can thus be considered a UV sex chromosome pair [39]. As described above, it has been proposed that anisogamy or oogamy could evolve through a size-regulatory gene becoming linked to an ancestral mating locus [11]. Both *MTM* and *MTF* haplotypes contain a putative cell-size regulatory gene, *MAT3*, whose alleles are highly dimorphic in sequence and expression between the sexes [20]. However, it is now apparent

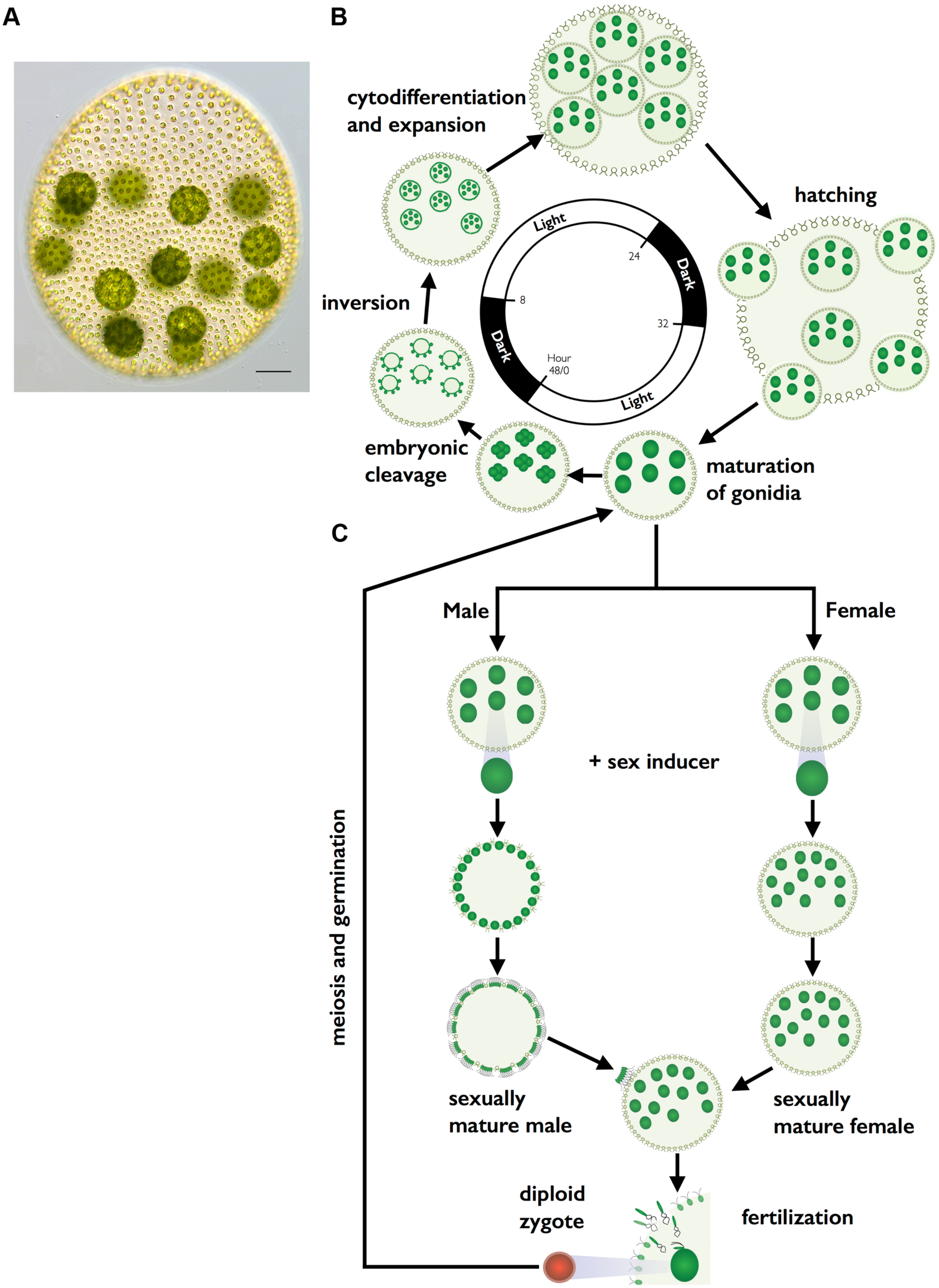
A
B
cytodifferentiation
and expansion
hatching
inversion
Light
Dark
Light
Dark
24
8
32
Hour
48/0
embryonic
cleavage
maturation
of gonidia
C
Male
Female
+ sex inducer
meiosis and germination
sexually
mature male
sexually
mature female
diploid
zygote
fertilization

Figure 1. *V. carteri* vegetative and sexual cycles. (A) Color DIC image of vegetative *V. carteri* spheroid with large reproductive cells (gonidia) on the interior and somatic cells on the exterior. Scale bar = 50 µm. (B) Key stages of the two-day vegetative reproductive cycle are depicted with relative timing indicated by the interior clock diagram showing the 16 h light and 8 h dark phases. Starting from ~6:00 and going clockwise vegetative gonidia undergo embryonic cleavage followed by inversion to make new juvenile spheroids. Juveniles grow and eventually hatch and mature into the next generation of parental spheroids. The vegetative cycle is identical for males and females. (C) Key stages of the sexual cycle are depicted top to bottom starting with vegetative male or female gonidia that have been exposed to sex-inducer. Sexually induced gonidia undergo modified embryogenesis to produce sexual males with 128 large androgonidia and 128 somatic cells, or sexual females with 32–48 eggs. Subsequent cleavage of androgonidia produces sperm packets that are released and swim to a female whereupon they dissolve into single sperm and enter the female spheroid to fertilize eggs. Diploid zygotes differentiate into environmentally resistant, orange-pigmented, dormant zygospores that when germinated undergo meiosis and produce three polar bodies plus a single haploid vegetative progeny that can reenter the vegetative reproductive cycle.
doi:10.1371/journal.pbio.1001904.g001

that anisogamy and oogamy in volvocine algae predate the appearance of *MAT3* allelic dimorphism in the lineage meaning that other mating locus genes probably underlie the origins of anisogamy and oogamy [40]. Although *MTM* contains a *MID* homolog, *VcMID* (Figure S1A), its role in sexual differentiation is unclear because *VcMID* mRNA is expressed constitutively in both vegetative and sexual stages of males [20]. The apparent uncoupling of *VcMID* expression from the sexual cycle suggests that the VcMid protein might have a function outside of the sexual cycle or that its function might be regulated differently than that of *CrMID* whose expression is induced by −N.

In this study, we tested the role of *VcMID* in *V. carteri* sex determination by making transgenic females that express VcMid protein or by knocking down its expression in males using RNAi. We found that expression of *VcMID* in females is sufficient to convert eggs to sperm packets, while its absence in males causes androgonidial cells to differentiate into eggs. However, alteration of VcMid expression did not affect female or male early embryonic patterning during which the number and location of germ-cell precursors is established. We found that *VcMID* mRNA is expressed in all cell types, but VcMid protein accumulation is regulated by cell type and its subcellular localization is restricted to nuclei of differentiating and mature male gametes. Swapping experiments with CrMid demonstrated that the VcMid DNA binding domain and N-terminal domain are both required for its function in directing spermatogenesis in *V. carteri*. Crosses with sex-reversed strains revealed sexually antagonistic interactions between genes in *MT* and the sexual development pathway controlled by VcMid that negatively impacted reproductive fitness when gamete type did not match the mating locus genotype.

Results

VcMid Controls Spermatogenesis

We tested the role of VcMid protein in sexual differentiation by generating female transgenic lines with an autosomally integrated *VcMID* transgene (pVcMID-BH) expressed under its own promoter and fused to a blue fluorescent protein (BFP) and a hemagglutinin (HA) epitope tag at its C-terminus to detect expression (*Eve::VcMID-BH*) (Figures 2A and S1B). Female transformants carrying an untagged version of *VcMID* (Figure S1C) had identical phenotypes as those carrying the tagged version, and all subsequent work was done with tagged strains and untagged transformants as a negative control for detection of VcMid protein. *Eve::VcMID-BH* lines showed a normal vegetative phenotype and constitutively expressed the mRNA for the *VcMID-BH* transgene, an expression pattern identical to the endogenous *VcMID* mRNA in males (Figure S2) [20]. As described above, when wild-type vegetative female gonidia are exposed to sex inducer they undergo modified embryogenesis and develop into sexual spheroids with 32–48 eggs and ~2,000 sexual somatic cells (Figure 2B). When wild-type vegetative male gonidia are exposed to sex inducer they undergo modified embryogenesis and develop with 128 sperm packets and 128 somatic cells (Figure 2C). When vegetative gonidia from *Eve::VcMID-BH* lines were exposed to sex inducer they developed into progeny spheroids with a novel pseudo-male sexual phenotype: They produced ~2,000 somatic cells and 32–48 sexual germ-cell precursors in a pattern similar to that of female eggs; but each of the 32–48 germ-cell precursors in the *Eve::VcMID-BH* lines underwent additional cleavage divisions like male androgonidia to produce sperm packets (Figure 2D). Moreover, the sperm produced in *Eve::VcMID-BH* lines were capable of fertilizing wild-type female eggs to produce characteristically orange-pigmented, thick-walled zygotes (Figure 2E). However, male function was incomplete as fertility defects were noted (see next section). Similar to wild-type sperm (and unlike wild-type female eggs) the *Eve::VcMID-BH* sperm cells were terminally differentiated and could not revert back to vegetative growth if left unfertilized. Another male-specific phenotype exhibited by *Eve::VcMID-BH* lines was frequent spontaneous occurrence of sexual differentiation in vegetative cultures [36], a trait whose underlying basis is not clear, but which appears to be under the control of *VcMID*.

Fertility Defects in Females Expressing VcMid

Although some of the *Eve::VcMID-BH* sperm were functional and could fertilize wild-type eggs, the sperm packets and sperm cells from these strains had multiple defects including heterochronic delays in maturation and hatching defects (Figure S3A). The sperm packets were four times larger than those from wild-type males and contained about four times as many sperm cells (256 sperm/packet) (Figure 2F and 2G), some of which were aberrantly formed in contrast with wild-type sperm cells that had uniform morphology (Figures 2H, 2I, and S3B–S3F). Nonetheless the crosses between *Eve::VcMID-BH* pseudo-males and wild-type females produced *MTF/MTF* diploid zygotes (Figure 2E) that could germinate and produce haploid progeny. Forty-one progeny from one such cross were genotyped, half of which (19/41) inherited the *VcMID-BH* transgene and developed as pseudo-males, and half of which lacked the transgene and developed as normal females (22/41).

Expression and Localization of VcMid Are under Post-transcriptional Control

The absence of a vegetative phenotype in *Eve::VcMID-BH* transgenic lines despite constitutive expression of the *VcMID-BH* mRNA (Figure S2) suggested that VcMid protein expression or localization might be under posttranscriptional control. Although we could not detect BFP fluorescence in *Eve::VcMID-BH* strains, we could detect the HA epitope tag by Western blotting at all stages of vegetative and sexual development (Figures 3A and S4). Using immunofluorescence (IF) a nuclear-localized signal was

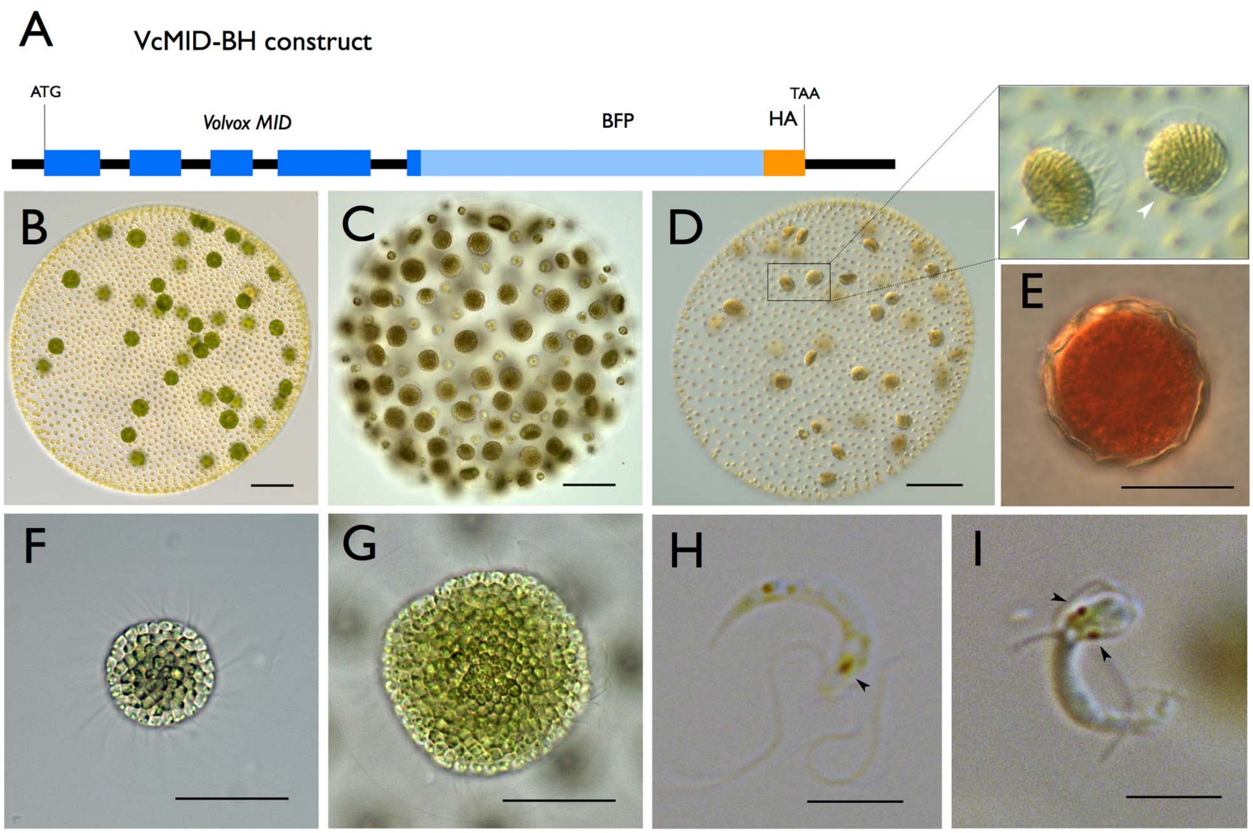

Figure 2. Ectopic Expression of *VcMID* converts egg precursors to sperm packets. (A) Diagram of *VcMID-BH* expression construct. Dark blue boxes depict *VcMID* exons; black lines depict introns and intergenic regions; light blue box depicts BFP; orange box depicts HA epitope. Start (ATG) and stop (TAA) codon positions are shown along with scale bar in black. (B) Wild-type mature sexual *Eve* (female) spheroid with ~32 large green eggs. (C) Wild-type mature sexual *AichiM* (male) spheroid with ~128 sperm packets. (D) Mature sexual *Eve::VcMID-BH* pseudo-male with ~32 sperm packets, two of which are magnified in expanded box to the right. Scale bars for (B–D) = 50 µm. (E) Mature zygote from *Eve::VcMID-BH*×*Eve*. (F) Sperm packet from wild-type *AichiM*. (G) Sperm packet from pseudo-male *Eve::VcMID-BH*. Scale bars for (E–G) = 25 µm. (H) Sperm cell from wild-type male with a single eyespot indicated by a black arrowhead. (I) Aberrant sperm cell from *Eve::VcMID-BH* pseudo-male with two eyespots indicated by black arrowheads. Scale bars for (H) and (I) = 5 µm.
doi:10.1371/journal.pbio.1001904.g002

detected for VcMid-BH protein in cleaving androgonidia and in mature sperm packets (Figures 3B–3J, S5A–S5R, and S6C–S6H), a result similar to earlier findings of Mid protein localization in sperm nuclei of *Pleodorina* [21]. However, nuclear VcMid-BH was not detected during early stages of sexual male embryogenesis prior to androgonidia cleavage (Figure S7).

We also examined VcMid-BH expression and localization in gonidia and somatic cells from vegetative spheroids. RNA was prepared from purified gonidial or somatic cells and reverse transcription and PCR (RT-PCR) detected *VcMID-BH* mRNA at similar levels in both cell types from transgenic females (*Eve::VcMID-BH*) and males (*AichM::VcMID-BH*) (Figure S8B and S8D). The endogenous *VcMID* transcript from wild-type males was also expressed in both vegetative cell types (Figure S8C and S8D). Whole cell extracts were prepared from purified *Eve::VcMID-BH* or *AichM::VcMID-BH* somatic cells and gonidia and subjected to SDS-PAGE and Western blotting to detect VcMid-BH protein (Figures 3K, S9A, and S9B). In contrast to *VcMID-BH* mRNA that was present in both vegetative cell types, VcMid-BH protein was only detected in vegetative somatic cells indicating that there is cell-type–specific regulation of VcMid protein synthesis or stability that restricts its accumulation to somatic cells during vegetative growth (Figures 3K and S6B). However, unlike the case for androgonidia and sperm cells, the VcMid-BH protein signal in vegetative somatic cells was excluded from the nucleus and was instead observed only in the cytosol and peri-nuclear region (Figures 3L–3O, S6I–S6P, and S9C–S9J). Together these data suggest that cell-type–limited expression and regulated nuclear localization of VcMid restrict its function to developing and mature sexual male germ cells in *V. carteri*.

VcMid Is Required for Spermatogenesis and Represses Oogenesis in Males

In order to test whether *VcMID* is necessary for sexual differentiation of males we developed a new strategy for gene knockdown on the basis of RNAi-inducing hairpin constructs. The hairpin-forming portion of the construct corresponding to *VcMID* sequences was inserted directly into the 3′ UTR of the *nitA* selectable marker gene to allow direct and simultaneous selection of both NitA+ and hairpin expression (Figures 4A and S10; Text S1). This strategy has been successful for other loci besides *VcMID* (unpublished data), but only the results for *VcMID* are presented

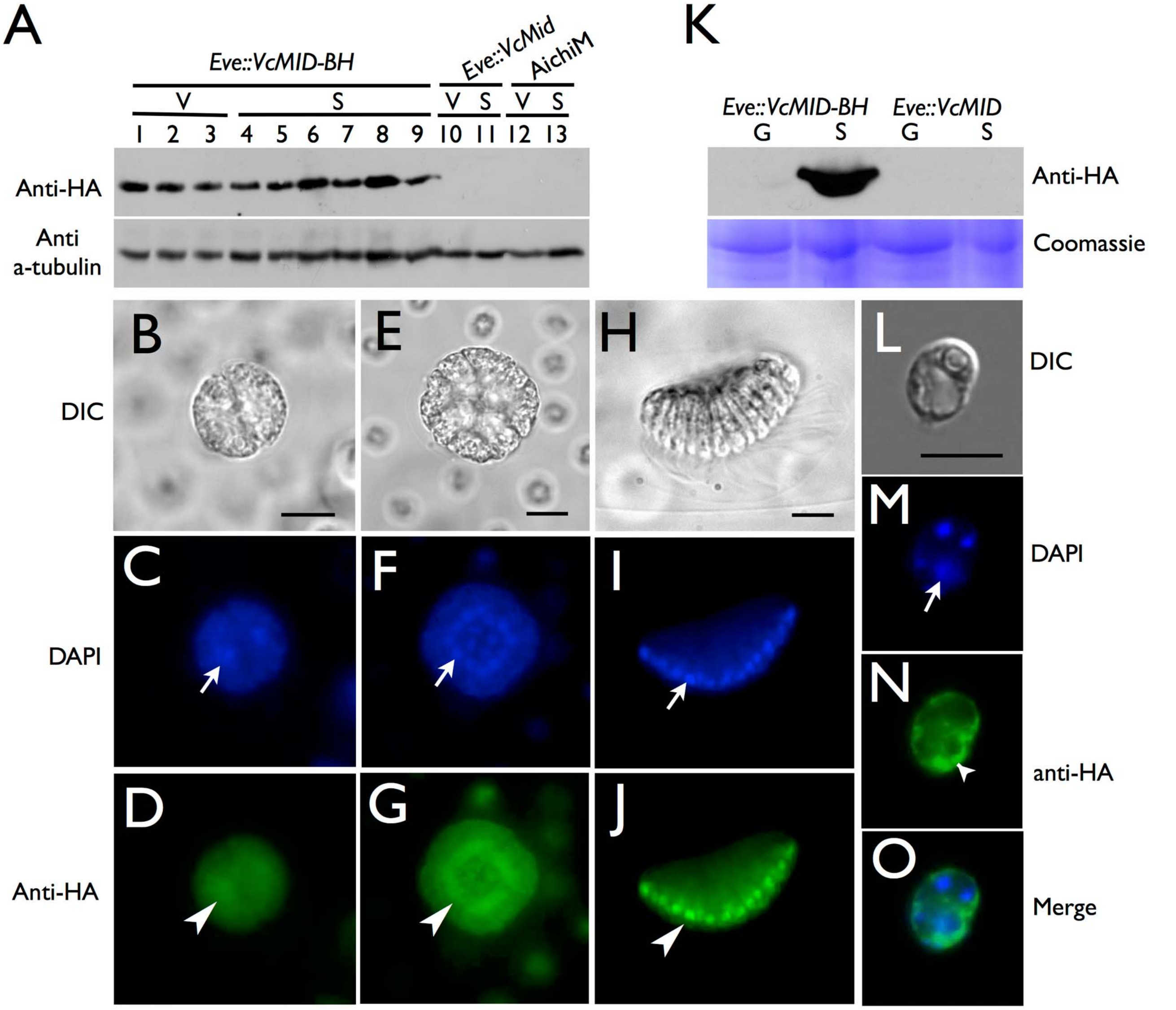

Figure 3. Cell-type restricted expression and sex-regulated nuclear localization of VcMid. (A) Immunoblot of SDS-PAGE fractionated protein extracts from HA-tagged pseudo-male strain *Eve::VcMID-BH* (lanes 1–9), untagged pseudo-male strain *Eve::VcMid* (lanes 10, 11), and wild-type male strain *AichiM* (lanes 12, 13). Lanes 1–3 contain extracts from vegetative spheroids at adult stage, mid-cleavage stage, and unhatched juvenile stage respectively. Lanes 4–9 contain extracts from spheroids undergoing sexual development with pre-cleavage stage, mid-cleavage stage, unhatched juvenile stage, cleaving androgonidia stage, and mature sexual adult stage, respectively. See also Figure S2B and S2C. Lanes 10 and 12 contain extracts from adult vegetative-stage spheroids. Lanes 11 and 13 contain extracts from mature sexual-stage spheroids. The bands in the upper panel are VcMid-BH protein detected with an anti-HA antibody. The bands in the lower panel come from the same blot re-probed with an anti-tubulin antibody as a loading control. (B–J) DIC (B, E, H) or false-colored IF images of cleaving androgonidia from *Eve::VcMID-BH* sexual germ cells at the two-cell stage (B–D), sixteen-cell (E–G) stage, and from a mature sperm packet (H–J). IF samples were stained with DAPI shown in blue (C, F, I) or with anti-HA shown in green (D, G, J). Arrows and arrowheads show locations of a representative nucleus from each image. Scale bars = 10 µm. (K) Upper panel, anti-HA immunoblot of SDS-PAGE fractionated protein extracts of purified vegetative gonidia (G) or somatic (S) cells from *Eve::VcMID-BH* and *Eve::VcMID* spheroids. Lower panel, Coomassie-stained gel used as a loading control. (L–O) IF detection of VcMid-BH protein from a representative vegetative somatic cell of *Eve:: VcMID-BH* stained as in (B–J). The arrows in (M, O) show the nucleus, while the chloroplast nucleoids (smaller DAPI-stained spots) are unlabeled. The VcMid-BH signal in (N, O) is excluded from the nucleus as evident in the merged image (O). Scale bar = 7.5 µm.
doi:10.1371/journal.pbio.1001904.g003

here. We also note that the *VcMID* knockdown phenotype was gene specific and did not occur when hairpins targeting other loci were introduced into *V. carteri* (unpublished data). Two hairpins targeting *VcMID* (*VcMID-hp1* and *VcMID-hp2*) were introduced into a *V. carteri* wild-type male strain *AichiM* to generate *AichiM::VcMID-hp1* and *AichiM::VcMID-hp2* transgenic lines (see Materials and Methods for details). All transgenic male strains had normal vegetative phenotypes, and both hairpin constructs reduced *VcMID* expression; but *AichiM::VcMID-hp1* lines had lower *VcMID* transcript levels than *AichiM::VcMID-hp2* lines (Figure S2). We note that unlike wild-type males or pseudomales (see above), vegetative cultures of *AichiM::VcMID-hp1* lines did not

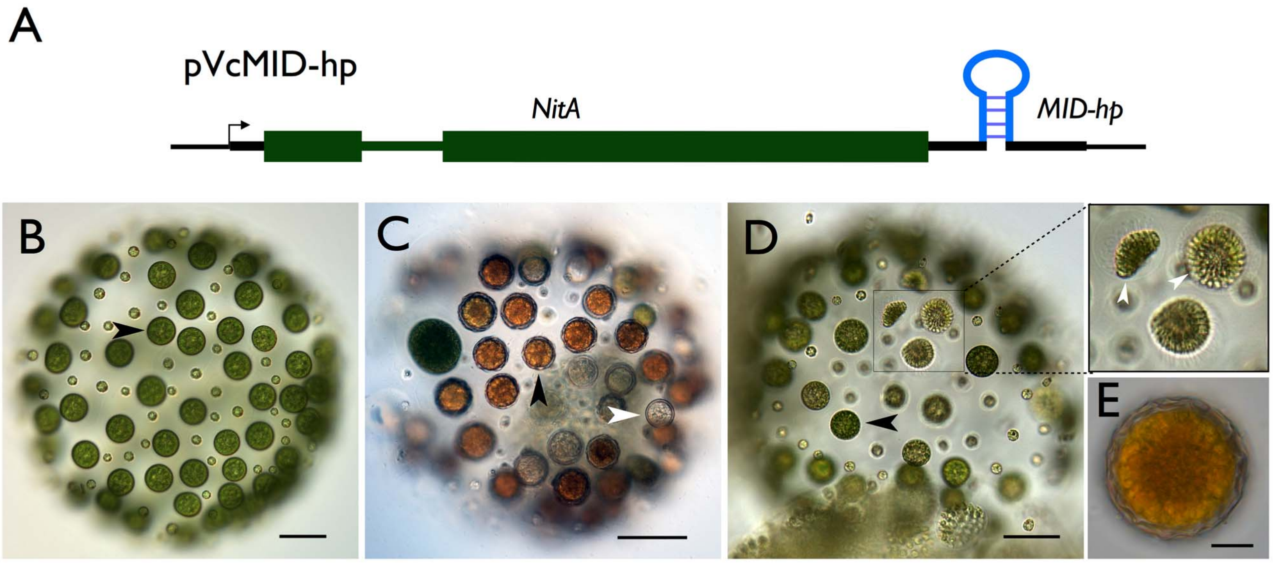

Figure 4. Conversion of male germ cells to eggs by knockdown of *VcMID*. (A) Diagram of *VcMID* hairpin construct. Green boxes, *NitA* exons; medium green line, intron; thick black lines, UTRs; *VcMID* hairpin structure inserted in 3′ UTR is shown in its approximate location. (B) Sexually induced *AichiM::VcMID-hp1* pseudo-female with eggs in place of sperm packets. Black arrowhead shows an egg. (C) *AichiM::VcMID-hp1* pseudo-female fertilized by wild-type male sperm. Black arrowhead shows a normal-appearing developed zygote; white arrowhead shows an aborted zygote. (D) Hermaphrodite phenotype of mature sexually induced *AichiM::VcMID-hp2* spheroid with eggs and sperm packets. Black arrowhead shows egg; boxed region has three sperm packets that are magnified in the expanded view to the right. Scale bars for (B–D) = 50 µm. (E) Zygote produced by self-fertilization of *AichiM::VcMID-hp2* spheroid. Scale bar = 10 µm.
doi:10.1371/journal.pbio.1001904.g004

undergo spontaneous sexual induction. Sexually induced *AichiM::VcMID-hp1* lines with strong knockdowns showed a novel phenotype: Their early sexual development proceeded as it would for a wild-type male strain and resulted in spheroids that contained 128 small somatic cells and 128 large cells that resembled uncleaved androgonidia (Figure 4B), but the large cells never underwent further cleavage into sperm packets. Instead, many of them could be successfully fertilized with wild-type male sperm to make *MTM/MTM* diploid zygospores (Figure 4C). The ability to differentiate as zygospores when fertilized indicates that the presumptive androgonidia in *AichiM::VcMID-hp1* strains were converted to functional eggs and that these strains were behaving as pseudo-females. However, unlike normal zygotes from a wild-type cross, 30%–50% of the zygotes from *AichiM×AichiM::VcMID-hp1* pseudo-female crosses died and bleached shortly after fertilization (Figure 4C), a phenotype that depended on addition of exogenous sperm. The surviving zygotes from these crosses produced some viable meiotic progeny, but germination and survival of the progeny were reduced compared with normal wild-type zygotes (Table S1). 29/43 viable progeny inherited the *VcMID-hp1* transgene and developed as pseudo-females, while 14/43 lacked the transgene and developed as normal males. The apparent deviation from a 1:1 inheritance pattern of the transgene was noted but was not pursued further in this study. The high mortality of pseudo-female eggs—whose mating loci are genetically male—suggest that *MTF* contains genes that promote female gamete and/or zygote fitness that are absent from *MTM*. If left unfertilized, the eggs from *AichiM::VcMID-hp1* lines could de-differentiate and reenter the vegetative reproductive cycle as do unfertilized female eggs. A similar phenotype as our pseudo-male strain was reported previously for a male mutant [41], but the mutant strain is no longer available for characterization.

Partial *VcMID* Knockdown Generates Self-Fertile Hermaphrodites

AichiM::VcMID-hp2 lines were not as severely knocked down for *VcMID* expression as *AichiM::VcMID-hp1* lines (Figure S2), and had a distinct hermaphrodite phenotype in which sexual spheroids developed with a mixture of normal-looking male sperm packets and pseudo-female eggs (Figure 4D). The hermaphrodite lines exhibited self-fertility as evidenced by zygospores that formed in sexually induced monocultures (Figure 4E). These results indicate that *V. carteri* sex determination is highly sensitive to VcMid dosage where either a male or female fate is established depending on the level of VcMid.

Volvox and *Chlamydomonas* Mid Proteins Are Functionally Distinct

In several instances genes from *C. reinhardtii* have been shown to function interchangeably with their *V. carteri* orthologs in developmental processes such as inversion and asymmetric cell division (reviewed in [42,43]). Mid proteins have at least two domains: The C-terminal region has a predicted RWP-RK motif DNA binding domain, while the N-terminal region does not show similarity to characterized protein domains from other organisms (Figure S1) [28]. We tested whether either of the domains from CrMid could substitute for those of VcMid to control spermatogenesis in *V. carteri*. To do so we generated three constructs in which all or part of the *CrMID* genomic coding region was substituted for *VcMID* sequences in pVcMID-BH. One construct contained the entire *CrMID* gene (pCrMID-BH) (Figure 5A) while the other two contained the *CrMID* N-terminal domain fused to the *VcMID* DNA binding domain (pMID-V_NC_C-BH) (Figure 5B), or the *VcMID* N-terminal domain fused to the *CrMID* DNA binding domain (pMID-C_NV_C-BH) (Figure 5C). All three

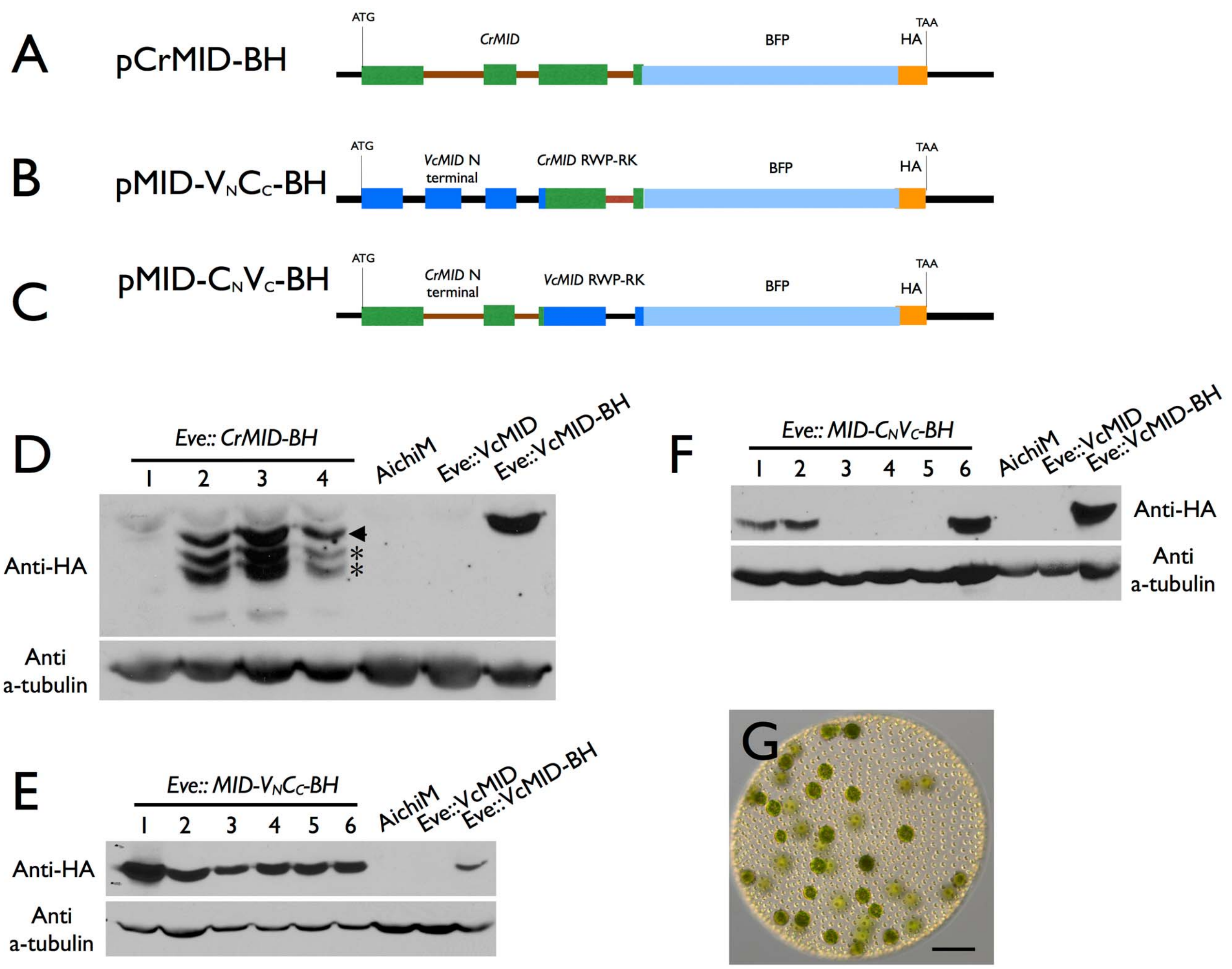

Figure 5. *Chlamydomonas MID* cannot substitute for *Volvox MID*. (A–C) Diagrams of constructs pCrMID-BH (A), pMid-V_NC_C-BH (B), and pMid-C_NV_C-BH (C). Dark blue boxes depict *VcMID* exons; black lines depict *VcMID* introns and intergenic regions; green boxes depict *CrMID* exons; brown lines depict *CrMID* introns; light blue boxes depict BFP; Orange box depicts HA epitope. Start (ATG) and stop (TAA) codons are shown along with scale bar in black. (D–F) Immunoblots of SDS-PAGE fractionated protein extracts from Eve transformants or control strains probed with anti-HA antibodies (upper panels) or re-probed with tubulin antibodies (lower panels). Numbered lanes indicate independent transformants, some of which express the tagged Mid constructs. Control strains are wild-type male AichiM, Eve transformed with untagged *VcMID*, and Eve transformed with tagged *VcMID-BH* shown in Figure 1A. (D) *Eve::Mid-CrMID-BH* transformants. Arrowhead indicates predicted full length CrMid-BH and asterisks represent breakdown or alternative processing products. (E) *Eve::Mid-V_NC_C-BH* transformants. (F) *Eve:: Mid-C_NV_C-BH* transformants. (G) Color DIC image of sexually induced *Eve::Mid-C_NV_C-BH* transformant number 6 showing typical arrangement of eggs similar to a wild-type female. Scale bar = 200 µm.
doi:10.1371/journal.pbio.1001904.g005

constructs as well as pVcMID-BH were introduced into wild-type females, and transformants that expressed the different predicted proteins were identified (Figures 5D–5F). Unlike *Eve::VcMID-BH* transformants that showed a pseudo-male phenotype (Figure 1D), none of the transformants that expressed CrMid or Mid chimeras had this phenotype, but instead always developed as wild-type females (Figure 5G; Table S2). The CrMid-BH protein was expressed as a full-length form and two shorter isoforms (Figure 5D), possibly due to proteolytic cleavage or incorrect pre-mRNA processing. However, lines with either of the two chimeric constructs expressed only full-length predicted proteins at levels comparable to VcMid-BH (Figure 5E and 5F). We conclude from these experiments that CrMid and VcMid are not functionally interchangeable, and that both the N-terminal and RWP-RK domains of VcMid are required to activate spermatogenesis in sexual germ cells.

Discussion

Deep Homology between Mating Types and Sexes

Although it was reasonable to predict that the route for evolving sexual dimorphism would be through addition of new genetic functions and pathways to a core mating locus as proposed originally by Charlesworth for the evolution of anisogamy [11], we found instead that a single conserved volvocine algal mating locus gene, *MID*, is largely responsible for controlling male versus female sexual differentiation in *V. carteri*. It is notable that *VcMID* controls multiple sexual traits in *V. carteri* that have no analogs in *C. reinhardtii*. The male traits controlled by *VcMID* include specialized cleavage divisions of androgonidia, sperm packet inversion, sperm packet hatching, specialized sperm cell morphology, and gamete recognition without flagellar adhesion.

The term deep homology is used to describe ancient and conserved genetic mechanisms that control traits that, on the surface, appear disparate or have no obvious homology relationship [44]. Metazoan eye development is a classic example; across phyla with very different eye architecture, it is controlled by the conserved transcription factor, eyeless/Pax6 [45]. In the case of volvocine algae Mid proteins control two very different manifestations of sexual reproduction in *C. reinhardtii* and *V. carteri* whose *MID* orthologs have been diverging for as long as 200 million years [46]. Sexually dimorphic gametes have evolved from isogamous ancestors several times in independent multicellular taxa, and the mechanism could be similar to, or different from, that in volvocine algae where a master regulatory gene acquired the ability to direct sperm-egg dimorphism. Oogamy was likely established very early in the Streptophyte lineage before the split between Charophyte algae and land plants, but the origins and bases of oogamy in Charophytes remain unclear [47]. The recent identification of an anisogamous sexual cycle in a Choanoflagellate—a unicellular relative of metazoans—introduces the potential for investigating the early evolution of gamete size dimorphism in animals [48].

MID Is a Master Regulator of Sex Determination in Volvocine Algae

Our results demonstrate for the first time a function for Mid protein in a volvocine algal sexual cycle outside of the genus *Chlamydomonas* and suggest that Mid could be the master regulator of mating type, gamete size, and gender throughout the volvocine lineage where *MID* genes have been identified in most genera [21,28,30]. Although *MID* sequences were previously shown to evolve rapidly, the finding that Mid protein from *C. incerta* (now reclassified as *C. globosa* [49]) can substitute for *C. reinhardtii* Mid indicates that functional conservation can be retained after speciation [27]. However, *C. reinhardtii* Mid protein could not substitute for the *V. carteri* ortholog, which appears to require both its native DNA binding and N-terminal domains to function in sex determination (Figure 5). Future work using cross-species complementation will help clarify whether Mid protein function coevolved with sexual dimorphism in volvocine algae as our data suggest might be the case.

Transcriptional regulatory network evolution has been studied in various developmental contexts [50–53], but very little is known about how regulatory networks are modified or coopted during unicellular to multicellular transitions [54]. The Mid system in volvocine algae represents a new opportunity for understanding how a cell-type specification pathway in a unicellular ancestor evolved to control a complex developmental program in a multicellular descendant. While mating-type differentiation in *Chlamydomonas* appears to involve differential expression of a small number of genes between the *plus* and *minus* gametes [14,22], a larger set of differentially expressed genes might be expected to specify sperm and eggs, which are developmentally very different from each other [37,55]. An important future goal will be to identify and compare the direct and indirect targets of Mid proteins in both *C. reinhardtii* and *V. carteri* that are predicted to be more numerous and diverse in *V. carteri*.

Another interesting question is whether *MID*-like genes function in sex determination in green algae outside of the volvocine lineage. The molecular bases for sex determination in most green algae and protists are poorly understood, but RWP-RK family proteins are found throughout the green eukaryotic lineage [56], including small Mid-like proteins in Prasinophyte algae [57], and even in distantly related Cryptophyte algae [58]. It remains to be seen whether these Mid-like proteins in non-volvocine species function in sex determination.

Evolution of Mid and Sexual Cycles in the Volvocine Lineage

As described in the Introduction, volvocine algae exhibit wide diversity in their sexual cycles: There are isogamous, anisogamous, and oogamous species with sexual cycles that can be heterothallic or homothallic. Among homothallic species some are dioecious (producing a mixture of all male and all female sexual offspring) or monoecious (producing sexual offspring containing gametes of both sexes in one individual) [16,55].

V. carteri is a heterothallic species with genetically determined sexes; but, a remarkable phenotype was produced by a partial RNAi knockdown of *VcMID* in males (Figure 4D). Rather than developing as male or female, the partial-knockdown male spheroids became self-fertile monoecious hermaphrodites that produced sperm and eggs in a single individual. On the basis of this observation, it can be inferred that sexual differentiation in *V. carteri* is bi-stable and highly sensitive to initial *MID* dosage. Once the Mid-sensitive step of development is initiated (which may coincide with nuclear translocation of VcMid protein), positive and negative feedback loops may be used to lock the sex determination program into a male or female state. This state could be achieved either independently of VcMid concentration, or through positive/negative reinforcement of initial expression states. The phenotype of hermaphroditic development by partial *VcMID* knockdown may be relevant to the evolution of homothallism, which appears to have arisen in all three major clades of *Volvox* [16,55,59]. We speculate that naturally evolved homothallic volvocine algae possess a *MID* gene whose expression is insufficient to specify 100% male gamete production—much like our *VcMID-hp2* strains. Moreover, the timing of *MID* expression in homothallic species of *Volvox* could play a role in determining monoecious versus dioecious reproductive development: If the Mid-sensitive developmental switch is triggered relatively late in development after germ-cell precursors are formed then a mixture of male and female gametes could develop within a single spheroid as we found with *VcMID-hp2* strains (i.e., homothallism, monoecy). In contrast, if the Mid-sensitive step occurred very early in development before individual germ cells were established, then the fate of all germ cells within a mature spheroid might be locked into a male or female program and the resulting population would produce a mixture of all-female or all-male spheroids (i.e., homothallism, dioecy).

The bi-stability of sex determination at intermediate levels of *MID* expression in *V. carteri* is also reminiscent of the *iso1* mutant phenotype in *C. reinhardtii* where *MT− iso1* cells differentiate into a mixture of *plus* and *minus* gametes that iso-agglutinate but cannot self-fertilize [60]. The self-infertility of *iso1 MT−* strains is due to the absence of *FUS1*, a gene from the *MT+* haplotype that is required for gamete fusion [61]. In contrast, there are no essential genes in *MTF* of *V. carteri* that are absolutely required for fertilization and subsequent germination of progeny from matings between pseudo-females (*VcMID* knockdown strains) and males. This lack of essential female *MT* genes for completing the sexual cycle may have facilitated transitions from heterothallism to homothallism in volvocine algae.

V. carteri Has Evolved New Regulatory Inputs for Mid

In volvocine genera other than *Volvox*—including the anisogamous genus *Pleodorina*— sexual differentiation and *MID* expression are both triggered by the absence of nitrogen (−N) [21,25,28,62]. In contrast, *V. carteri f. nagariensis* and other *Volvox* species use species-specific pheromones called sex inducers to trigger sexual differentiation [16,55,63]. A seemingly parsimonious evolutionary route for rewiring input into the Mid pathway in *V. carteri* would simply place *VcMID* transcription under the control of sex inducer

instead of nitrogen availability. Unexpectedly, however, *VcMID* mRNA and VcMid protein are both expressed constitutively at all life cycle stages (Figures 3A and S4) [20], and unlike the case in *C. reinhardtii*, VcMid appears to be under at least three types of posttranscriptional control: (i) Although *VcMID* mRNA is present in both vegetative cell types (somatic and gonidial cells), VcMid protein is only translated or stably produced in somatic cells and is absent from vegetative gonidia and vegetative embryos (Figures 3K and S6B). (ii) The VcMid protein produced in somatic cells is excluded from the nucleus (Figures 3L–3O, S6I–S6P, and S9C–S9J). (iii) In response to the presence of sex inducer, VcMid protein accumulates in the nuclei of cleaving androgonidial cells and in the nuclei of sperm cells where it is presumed to function in specifying sperm development (Figure 3B–3J, S5, and S6C–S6H).

In depth study will be required to determine how cell-type–regulated production and localization of VcMid are achieved, but it seems reasonable to infer that one or more factors are produced in sexually induced spheroids that promote the translation and/or stability of VcMid and its nuclear localization in androgonidia and sperm. The factor may interact directly with VcMid as a partner and may help specify its localization at promoters of target genes, but this idea remains to be tested. The absence of VcMid protein in vegetative gonidia despite its message accumulating to the same extent as in somatic cells seems puzzling at first glance. However, the block in stability or translation of VcMid in gonidia may have evolved as a failsafe mechanism to prevent sexual differentiation during vegetative embryogenesis. Such a mechanism would be unnecessary in vegetative somatic cells that are already terminally differentiated, but could potentially be important for vegetative gonidial cells because male sexual differentiation is irreversible and would be fatal if it occurred at the wrong time. However, why vegetative somatic cells express VcMid remains a mystery. As noted in Results, we observed no obvious vegetative phase phenotypes in *AichiM::VcMID-hp* strains whose somatic cells were missing VcMid, or in *Eve::VcMID-BH* strains that contained VcMid in somatic cells, but a definitive conclusion about whether cytoplasmic VcMid has a role in vegetative somatic cells awaits more in depth examination.

Uncoupling of Gender from Sex Chromosome Identity Uncovers Possible Sexually Antagonistic Interactions in the *MT* Locus

Our results show that the presence or absence of *VcMID* is the key determinant of differentiation in *V. carteri* sexual spheroids; yet the *MT* locus of this alga has around 70 additional genes, many of which show sex-biased gene expression [20]. Some of the *MT* genes are present only in the male or only in female haplotype, while the majority are male and female gametolog pairs that are highly diverged in sequence and expression pattern [20]. Our ability to uncouple gender from sex chromosome identity by manipulation of *VcMID* expression allowed us to uncover potential contributions of male and female *MT* genes to sexual dimorphism and reproductive fitness. Haploid sex chromosome systems have received less attention than diploid systems, but there are several predictions about them that our results begin to address. Under haploid dioecy, recessive mutations in sex-linked genes are not sheltered from selection as they are in the heterogametic sex of diploid systems, and are therefore expected to degenerate equally and lose only genes required for the opposite sex and not their own [64]. We note, however, that in *V. carteri MT* there is no evidence for an allele of a gametolog pair having been eliminated from one mating haplotype and retained in the other [20]. Haploid sex chromosomes are predicted to be similar to diploid systems in that both should accumulate sexually antagonistic alleles that benefit one sex, but harm the other [39,65], and should accumulate repeat sequences, as appears to have occurred in both *Volvox* and in the bryophyte *Marchantia* [20,66]. Accumulation of sexually antagonistic alleles has not been tested for haploid sex chromosomes, but the extensive divergence between *V. carteri MT* gametologs suggests that this phenomenon may contribute to the developmental and fitness defects we observed in pseudo-male and pseudo-female strains.

Although none of the sex-limited *MT* genes besides *VcMID* appear to be essential for *V. carteri* sex determination and completion of the sexual cycle, they clearly impact sexual development and reproductive fitness. A striking phenotype for both the pseudo-male and pseudo-female strains was the patterning of their germ-cell precursors formed during sexual development (Figures 2B–2D and 6A–6C). It is clear from these phenotypes that sexual germ cell patterning (i.e., the number and distribution of germ-cell precursor cells and ratio of germ-cell precursors to sexual somatic cells) is separable from germ cell differentiation and is not controlled by the VcMid pathway. Instead, this sexual patterning trait must be controlled by other *MT* genes (Figure 6). One candidate for this male-female patterning difference is the *MAT3* gene that encodes the retinoblastoma-related homolog in *V. carteri* [20,67]. In *Chlamydomonas*, Mat3 protein controls the multiple fission cell cycle by establishing the threshold size at which division can occur and by coupling the extent of cell division to mother cell-size [68,69]. Although not directly involved in determining gamete cell-size as we had originally predicted [40], it is possible that the male and female alleles of *VcMAT3* dictate the timing of asymmetric cell divisions by coupling embryonic blastomere cell-size to the asymmetric division machinery. Future work will be aimed towards determining the role of male and female VcMat3 gametologs in sexual cell division or other parts of the *V. carteri* sexual cycle.

In addition to defects in germ cell patterning in *Volvox* pseudo-males and pseudo-females, these strains show other reproductive defects. These include abnormal sperm cell shape and morphology (Figures 2H, 2I, and S3B–S3F), low efficiency of sperm packet hatching (Figure S3D), and delayed timing of androgonidial cleavage into sperm packets (Figure S3A). In depth study may reveal other defects in pseudo-male sperm related to cytoskeletal organization, gamete recognition, motility, and fertilization dynamics. Although egg cells lack distinct morphological features like sperm, we noted a very high mortality rate in pseudo-female eggs that occurred when we attempted fertilization. The mortality we observed under these conditions could be due to pre-zygotic defects in the eggs caused by their smaller size than wild-type female eggs or by a mismatch between the male mating locus and the female sexual differentiation pathway. The mortality might also be due to zygotic defects that occur when a copy of the female mating locus is absent from the zygote immediately after fertilization. Future work aimed at developing quantitative assays for distinct steps of fertilization will allow us to document in more detail the fitness contributions of male and female *MT* genes to the sexual cycle.

Materials and Methods

Detailed Materials and Methods are provided in Text S1. Materials used in this study will be made available upon request with the completion of a Materials Transfer Agreement from Donald Danforth Plant Science Center.

Volvox Strains and Culture Conditions

Eve (*Volvox carteri. f. nagariensis* UTEX 1885) and *AichiM* (*Volvox carteri. f. nagariensis* NIES 398) were obtained from stock centers http://web.biosci.utexas.edu/utex/ and http://mcc.nies.go.jp/,

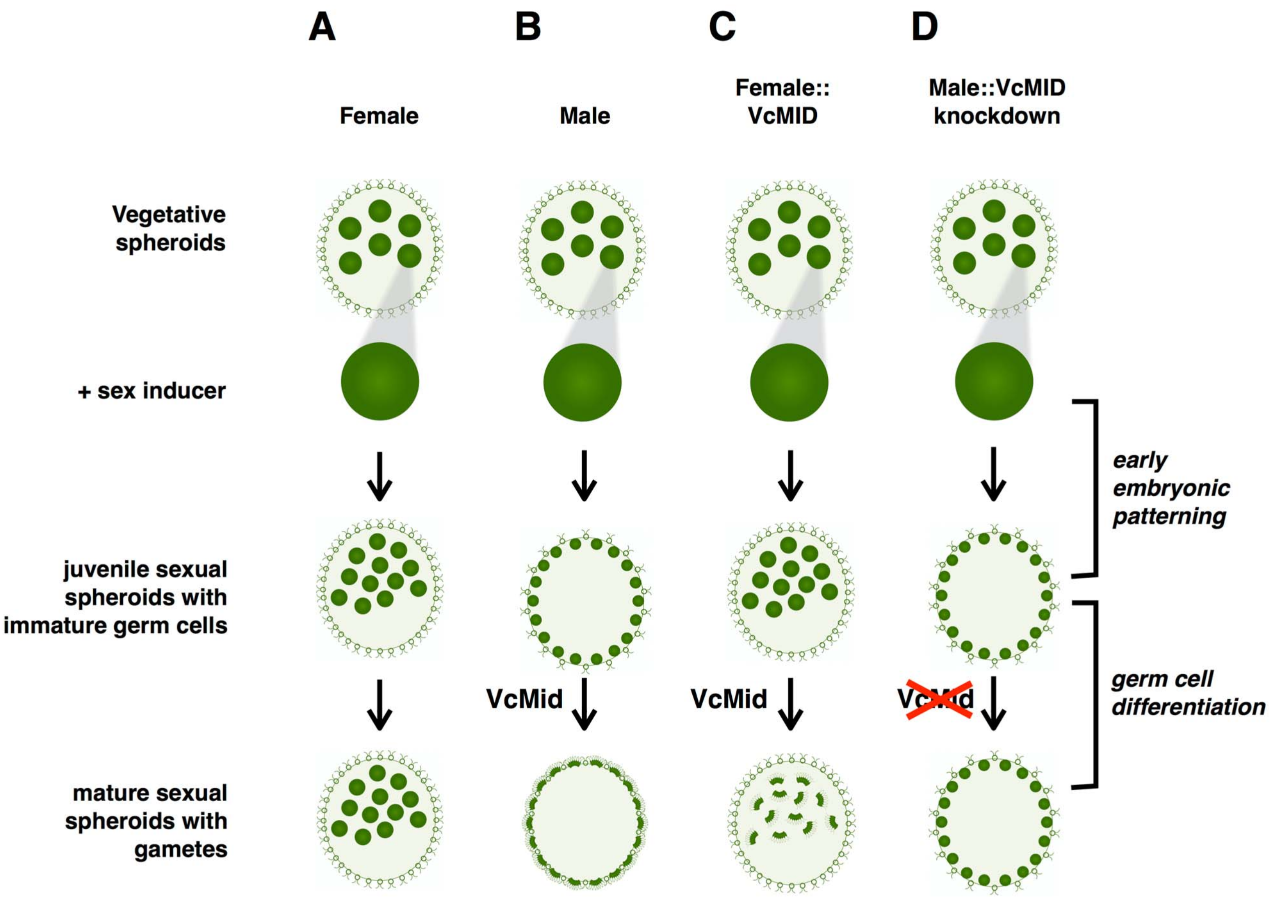

Mating locus	*MTF*	*MTM*	*MTF*	*MTM*
Mid	-	+	+	-
Early embryonic patterning	female	male	female	male
Germ cell differentiation	female	male	male	female
phenotype	female	male	pseudo-male	pseudo-female

Figure 6. Summary of the roles of *VcMID* and the mating locus in sexual differentiation. The upper section shows the process of sexual differentiation, top to bottom, starting from vegetative spheroids that are treated with sex inducer. The fate of a single sexually induced gonidia is shown in each case with its phenotype after embryogenesis and after sexual differentiation. Labels to the left show the stages depicted. Labels to the right show the developmental events that are controlled independently by the mating locus (early embryonic patterning) and the VcMid pathway (germ cell differentiation). (A–D) The four columns depict the fates of wild-type females (A), wild-type males (B), females expressing VcMid (pseudo-males) (C), and males with VcMid knocked down (pseudo-females) (D). The table below summarizes mating locus genotype, early embryonic patterning, VcMid expression, germ cell differentiation, and phenotypic outcome for each of the four strains. Male and female patterns are colored in blue and red, respectively.
doi:10.1371/journal.pbio.1001904.g006

respectively. The strains that were used for transformations are described below and in Text S1. Other strains are described in Table S3. Growth of strains was in standard Volvox medium (SVM) or urea-free standard Volvox medium (UF-SVM) with growth conditions described in more detail in Text S1.

Plasmid Construction

All plasmid constructs were made using standard molecular cloning methods and manipulations [70]. PCR amplifications used for plasmid construction were done with Phusion Polymerase (Thermo Scientific) according to the manufacturer's

guidelines (see also PCR amplification conditions below). Primers used in this study are described in Table S4.

Nuclear Transformation of *V. carteri* and Generation of Transgenic Strains

Constructs were introduced into *NitA*− female strain *E15* or male strain *A18*. Transformation or co-transformation of *E15* or *A18* with nitrate-reductase (*NitA*) encoding plasmid pVcNR15 [71], pVcMID hp1, or pVcMID hp2 was done as previously reported [72] with minor modifications described in Text S1. Transformed gonidia cells were selected in UF-SVM.

Induction of Sexual Development and Phenotypic Scoring

For each assay three vegetative juvenile-stage spheroids were placed in a well of a six-well microtiter plate with ~9 ml SVM per well and 10 μl of sex inducer with a titer of 10^6, and maintained at 32°C in a 16 h:8 h light:dark cycle [34]. The phenotypes of the 40–50 sexual progeny spheroids that resulted from sexual development of gonidia in the three starting spheroids were scored visually under a dissecting microscope and documented using a compound light microscope (Leica DMI6000B, 40× objective, differential interference contrast [DIC] optics) after 3–7 days.

Mating

Mating and zygote germination were performed as previously described with minor modifications [73]. Parental strains were grown to a density of ~300 unhatched juveniles in 350 ml of SVM at which point sex-inducer was added. In the subsequent cleavage cycle sexual spheroids were produced and allowed to mature. Egg-bearing females were released from their parental spheroid by gentle pipetting 3–5 hours prior to hatching and mixed with males that had their sperm packets released from their vesicles within the parental spheroid by a similar procedure. Matings took place in a glass 150 mm×25 mm petri dish at a density of 10–15 spheroids/ml. The petri dish was placed on a light box within a 30°C growth chamber for 8–16 hours, and then the dishes were wrapped with aluminum foil and left at 32°C for at least one week and typically for three weeks prior to germination.

Zygote Germination

Drawn-out Pasteur pipets were used to manipulate zygotes. In order to remove residual sex-inducer and any other potential inhibitors of germination, zygotes were put into 1.5 ml tubes with ~1 ml SVM medium and washed as follows: Tubes were vortexed for 10 min, then spun briefly at ~1,000 rpm in a microcentrifuge to pellet zygotes. The supernatant was removed and the washing step was repeated three times. The washed zygotes were transferred to a sterile glass depression slide, washed briefly with SVM, and allowed to settle to the bottom. Ten to 50 zygotes were transferred to a sterile glass depression well containing SVM with 60 μg/ml carbenicillin, and incubated in a 16 h:8 h light:dark 30°C growth chamber. Zygotes germinated after two to six days. The germling colonies were individually transferred to six-well microtiter plates for growth and clonal expansion.

RNA Preparation, cDNA Preparation, and Semi-quantitative RT-PCR

Total *Volvox* RNA was prepared as previously described [20]. cDNA was prepared from 5 μg total RNA following the manufacturer's protocol for Thermoscript (Invitrogen) using a 10:1 mixture of oligo dT and random hexamer for priming and the following cDNA synthesis reaction temperatures: 25°C 10′, 42°C 10′, 50°C 20′, 55°C 20′, 60°C 20′, 85°C 5′, after which the reactions were treated with RNaseH. Reactions were diluted 1:10 with 10 mM Tris pH 8.0, 1 mM EDTA (TE), and stored at −20°C. *S18* and *VcMID* expression was measured using amplification with primer sets *VcMid*.f1 and *VcMID*.r1 and S18.1 and S18.2 as described previously [20] and in Table S4.

Cell Separation

Vegetative gonidia and somatic cell separation was done by mechanical disruption and differential centrifugation. Cultures grown in four 350 ml SVM flasks with ~5,000 spheroids/flask were collected with a magnetic filter funnel with 25 μm nylon mesh filter, and transferred to a 40 ml Kimble Kontes Dounce homogenizer. Spheroids were broken with a tight-fitting pestle (B type) with six strokes. Broken spheroids were transferred to a 50 ml Falcon tube, and the volume adjusted to ~40 ml with SVM, after which 2.8 ml Percoll was added and the tubes spun at 200*g* for 5 min at room temperature. The supernatant containing somatic cells was transferred to a beaker and diluted to 200 ml with SVM. The pellet containing gonidia or embryos was washed two times with 50 ml SVM and gonidia collected after each wash by centrifugation at 200*g* for 5 minutes. Pure gonidia were then collected in a filter funnel using 10 μm nylon mesh, which allows any remaining somatic cells to pass through. To obtain pure somatic cells the diluted supernatant from the Percoll step above was spun at 460*g* for ~3 min to pellet any contaminating gonidia. The low speed spin supernatant was then spun at 3,220*g* for 5 min to obtain a pure somatic cell pellet, which was washed twice with 50 ml SVM prior to extraction of RNA or protein.

Western Blotting

Approximately 1,000 synchronized spheroids at designated stages were hand picked for protein sample preparation. Pelleted spheroids were mixed 1:1 with Volvox Lysis Buffer (1× PBS supplemented with 1% NP40 [IPEGAL], 1× Sigma Plant Protease Inhibitor Cocktail [catalog number P9599], 5 mM PMSF, 10 mM benzamidine, 5 mM EDTA, 5 mM EGTA). Spheroids and cells were disrupted using a Covaris S220 ultrasonicator according to the manufacturers instructions with the following program settings: PP = 200, DF = 20, CpB = 300, T = 6°C, and t = 300 s in TC 12×12 tubes at 4°C. After lysis the samples were centrifuged at full speed in a microfuge to pellet debris and the supernatant mixed with sample buffer and boiled prior to gel fractionation. SDS-PAGE and Western blotting were performed using standard procedures [70]. SDS-PAGE gels were blotted to Immobilon-P PVDF membranes (Millipore) prior to immunodetection. The rat monoclonal antibody 3F10 (Roche) was used for detection of the HA epitope on immunoblots and for IF. Tubulin was detected with an anti-α-tubulin antibody purchased from Sigma-Aldrich (clone B-5-1-2, catalog number T6074). Antibodies were used at the following dilutions: 250 mg/ml anti-HA, 1:2,000; and 2 mg/ml anti-α-tubulin, 1:20,000, all diluted in PBS with 0.05% Tween 20. Blocking was performed with 40 ml of 5% nonfat dry milk in PBS with 0.05% Tween 20 for one hour. Blots were incubated overnight at 4°C min (anti-HA antibody). Blots were washed three times with PBS and 0.05% Tween 20 at room temperature for 10 min, then horse radish peroxidase (HRP)-conjugated goat anti-rat secondary antibody (Thermo Scientific) was used at 1:2,500 dilution and incubated with blots at room temperature in PBS, 5% nonfat dry milk, 0.05% Tween 20 for 1 h. Blots were then washed three times with PBS and 0.05% Tween 20 at room temperature for 10 min and then briefly rinsed with deionized water. Antigen was detected by

chemiluminescence (Luminata Forte Western HRP Substrate, Millipore) using autoradiographic film (HyBlot CL autoradiography film, Denville Scientific Inc.)

Immunofluorescent Staining and Microscopy

Eve::VcMID-BH or *Eve::VcMID* (as a negative control) samples were processed in parallel and imaged under identical conditions. Spheroids were collected using a magnetic funnel with a 25 µm nylon mesh filter (Pall Scientific). Sexual spheroids were fixed with 2% or 4% paraformaldehyde with 1× plant protease inhibitor cocktail (Sigma) (10 µl/ml), 1 µm ALLN (catalog number 208719, VWR International), 1 µm MG132 (catalog number 133407-82-6, Cayman Chemical), 1 mM dithiothreitol (DTT) for 1 h on ice. Spheroids or cells were then washed with PBS and resuspended in cold methanol (−20°C) for five minutes. The methanol wash was repeated two more times and then samples were washed again with PBS and rehydrated in PBS at room temperature for 15 min after which they were adhered to poly-L-lysine–coated cover slips. Cover slips were blocked for 30 min in blocking buffer (5% BSA and 1% cold-water fish gelatin) and incubated for 30 min in the same buffer with 10% (v/v) normal goat sera (Antibodies Incorporated). Cover slips were incubated overnight in anti-HA 1:500 (1:300 for somatic cell staining) in 20% blocking buffer at room temperature or at 4°C and washed 6×10 min with 20% blocking buffer in PBS 0.05% Tween 20. After washing out unbound primary antibody cover slips were incubated with AlexaFluor 488 conjugated goat anti-mouse secondary antibodies (Invitrogen) 1:500 (1:300 for somatic cells staining) in 20% blocking buffer and incubated for 1 h at room temperature (for 4 h at 4°C for somatic cells staining) in the dark. Following six washes with 20% blocking buffer diluted in PBS and 0.05% Tween20, the cover slips were incubated for 15 min in 2 µg/ml DAPI and then washed for 5 min in PBS. Excess liquid was removed, and the cover slips were mounted with VectaShield (Vector Labs) or Mowiol:PPD (PPD = p-phenylenediamine 1,4-Benzenediamine hydrochloride [Sigma P1519]) with a 9:1 ratio. Microscopy was performed with a Leica DMI6000 B using DIC optics or using the following filter cube sets and illumination with a Prior Lumen 200 light source: A4: excitation BP 360/40; dichroic 400; emission BP470/40, L5: excitation BP 480/40; dichroic 505; emission BP 527/30. Where indicated Z stacks were subject to deconvolution using the Leica Advanced Fluorescence Application Suite. Images are representative of results from at least three independent experiments.

Supporting Information

Figure S1 Mid protein alignment and diagrams of pVcMID and pVcMID-BH constructs. (A) Multiple sequence alignment of Mid orthologs from Volvocine algae generated by MUSCLE [74]. The blue line above the sequences demarcates the RWP-RK domain. Species abbreviations are Cr, *Chlamydomonas reinhardtii*; Ci, *Chlamydomonas incerta*; Gp, *Gonium pectorale*; Ps, *Pleodorina starrii*; Vc, *Volvox carteri*. Sequences and alignments were described previously [20,30]. The orange triangle marks the junction point between the N-terminal domain and RWP-RK domain of Mid chimeras described in Figure 5. (B) pVcMID-BH and (C) pVcMID plasmid constructs. Blue filled boxes, exons; black lines, non-coding regions; light blue box, BFP coding sequence; orange box, tandem hemagglutinin (2× HA) epitope tag. Locations of Start (ATG) and termination (TGA) codons as well as relevant restriction enzyme sites are shown. Scale bar is in upper right.
(TIF)

Figure S2 *VcMID* expression in wild-type and transgenic strains. Gel images from semi-quantitative RT-PCR show *VcMID* expression levels in indicated strains. RNA from mature vegetative (veg) or mature sexual (sex) spheroids was used for cDNA synthesis and amplification with *VcMID* primers or with ribosomal protein gene *S18* primers as an internal control. Reactions were stopped at the indicated cycle numbers below or above each lane and used for agarose gel electrophoresis followed by ethidium bromide staining and visualization. A negative control reaction without added template was included in each experiment and loaded in the far right lane for each set of reactions.
(TIF)

Figure S3 Sperm development in wild-type male strain *AichiM* and pseudo-male strain *Eve::VcMid-BH*. (A) The diagram shows a comparison of the developmental chronology for spermatogenesis in wild-type male (*AichiM*) and pseudo-male (*Eve::VcMid-BH*) strains. Dark and light boxes in the middle depict successive diurnal cycles (16 h light:8 h dark). The developmental chronologies start with newly formed, post-embryonic juvenile sexual spheroids (far left). In both strains the timing of development up to this stage is the same. The upper sequence depicts a wild-type male whose androgonidia begin dividing about one day post-embryogenesis. Fully mature sperm packets are formed by the end of the second dark cycle. The sperm packets are released from their parental vesicle and eventually dissociate into individual sperm cells by the middle of the next light cycle. The lower part of the panel shows the same sequence of events for pseudo-male strains whose androgonidia take a full extra day to begin dividing into sperm packets, and whose fully formed sperm packets are delayed in hatching from the parental vesicle. Scale bars for juvenile spheroids = 20 µm, early cleaving androgonidia = 10 µm, mature sperm packets = 25 µm, released sperm = 5 µm. (B) Dissociating sperm packet with wild-type mature sperm from *AichiM*. Black arrowhead shows a single mature sperm cell. Scale bar = 10 µm. (C) Two individual wild-type sperm cells from *AichiM* at higher magnification. Scale bar = 5 µm. (D) Dissociating mature sperm packet from *Eve::VcMID-BH* pseudo-male. The location of the mostly intact vesicle wall surrounding the sperm packet that would normally be dissolved in wild-type males is shown with a dashed line. Black arrowheads indicate cells with relatively normal elongated sperm-like morphology. Black arrows indicate rounded non-sperm-like cells that are only observed in sperm packets of pseudo-males. Scale bar = 20 µm. (E) and (F) Single sperm cells from *Eve::VcMID-BH* pseudo-males. The sperm cell in (E) is abnormally large while the sperm cell in (F) has a mislocalized eye-spot in the center rather than apical end of the cell. Scale bar = 5 µm.
(TIF)

Figure S4 Vegetative and sexual development of *Eve::VcMid-BH* with sampling time points. (A) Vegetative life cycle diagram of *V. carteri*. The inner ring shows light (open bar) and dark (closed bar) periods in the 48 hour reproductive cycle. Numbers 1, 2, and 3 show stages at which extracts were prepared for Western blotting in Figure 3A. Images depict key stages starting with mature spheroids at ~10 o'clock (1) and proceeding clockwise to show cleavage stage (2), pre-inversion stage, early juvenile stage, adult stage (3), and hatching. (B) Diagram showing *Eve::VcMid-BH* sexual differentiation. Light and dark phases are shown with open and closed bars. Numbers 4–9 show stages at which extracts were prepared for Western blotting in Figure 3A. Diagrammed from left to right are pre-cleavage stage (4), cleavage stage (5), juvenile stage (6), cleaving androgonidia stages (7, 8), and mature stage with sperm packets with a spheroid that has hatched

from its parent (9). Images below 6–9 are expanded views of maturation for a single androgonidia showing cleavage into a sperm packet.
(TIF)

Figure S5 VcMid-BH sub-cellular localization during sperm development. IF images from Figure 2 along with negative control images for each stage. (A–R) Images of two-cell androdonidia (A–F), 16-cell androgonidia (G–L), and mature sperm packets (M–R) from HA-tagged Mid-expressing strain *Eve::VcMID-BH* (A, B, C, G, H, I, M, N, O) and untagged control transgenic strain *Eve::VcMID* (D, E, F, J, K, L, P, Q, R). Cells at each stage were imaged by DIC light microscopy (A, D, G, J, M, P), by DAPI fluorescence to visualize DNA in blue (B, E, H, K, N, Q), and by indirect IF to detect the VcMid-BH signal in green (C, F, I, L, O, R). Arrows indicate representative nuclei and arrowheads indicate nuclear staining of VcMid. Scale bars = 10 µm.
(TIF)

Figure S6 Cell-type restricted expression and sex-regulated nuclear localization of VcMid in wild-type males. (A) Immunoblot of SDS-PAGE fractionated protein extracts from independent *AichiM::VcMID-BH* transformants (lanes 1–7), wild-type male strain *AichiM*, and untagged pseudo-male strain *Eve::VcMid* (lanes 8, 9). The bands in the upper panel are VcMid-BH protein detected with an anti-HA antibody. The bands in the middle panel come from the same blot stained with Ponceau S as a loading control. Lower panel, Coomassie-stained gel with equivalent extract volumes loaded as for Western blot gel. (B) Upper panel, anti-HA immunoblot of SDS-PAGE fractionated protein extracts of purified vegetative gonidia (G) or somatic (S) cells from *AichiM::VcMID-BH* transformant number *4*, wild-type male strain *AichiM*, and untagged pseudo-male strain *Eve::VcMid*. The middle and lower panels are the same as in (A). (C–P), DIC (C,F,I,M), or false-colored deconvolved IF images of sperm packet from *AichiM::VcMID-BH* (C–E) and *AichiM* (F–H), and of vegetative somatic cell from *AichiM::VcMID-BH* transformant *number 7* (I–L) and vegetative somatic cell from *AichiM* (M–P). IF samples were stained with DAPI shown in blue (D, G, J, N) or with anti-HA shown in green (E, H, K, L, O, P). The VcMid-BH signal in (K) is mostly outside the nucleus as evident in the merged image (L). Arrows and arrowheads show locations of a representative nucleus from each image. Scale bars = 10 µm in (C–H), and scale bar = 7.5 µm in (I–P).
(TIF)

Figure S7 VcMid is not detectable in nuclei of *Eve::VcMID-BH* embryos undergoing sexual development. DIC (A, E) and fluorescent (B–D, F–H) images of eight or 16 cell stage embryos from *Eve::VcMID-BH* (A–D) or control *Eve::VcMid* (E–H) transformants. DAPI staining (B, F) is false colored blue with representative nuclei indicated by arrows. Anti-HA immunostaining (C, G) is false colored green. Merged DAPI and anti-HA images are in (E, H). No nuclear signal for VcMid-BH is detectable above background staining. Scale bar = 10 µm.
(TIF)

Figure S8 *VcMID* and *VcMID-BH* mRNA expression in purified gonidia and somatic cells from *V. carteri* males. (A) pVcMID-BH diagram showing the location of primers used to amplify the *VcMID-BH* transgene (*VcMID*cDNA.f1 and *BFP*-r) and the primers that amplify both the endogenous *MID* gene and *VcMID-BH* transgene (*VcMID*cDNA.f1 and *BFP*-r). (B–D) Gel images from semi-quantitative RT-PCR show *VcMID* and/or *VcMID-BH* expression levels in indicated strains. RNA from gonidia or somatic cells was used for cDNA synthesis and amplification with *VcMID* primers or with ribosomal protein gene *S18* primers as an internal control. Reactions were stopped at the indicated cycle numbers below or above each lane and used for agrose gel electrophoresis followed by ethidium bromide staining and visualization. A negative control reaction without added template was included in each experiment and loaded in the far right lane for each set of reactions.
(TIF)

Figure S9 VcMid-BH cell-type expression and subcellular localization in vegetative phase *V. carteri*. (A) and (B) Microscopic images of purified vegetative gonidia (A) and somatic cells (B) from *Eve:: VcMID-BH* used for Western blot detection of VcMid. Scale bars, 100 µm in (A) and 200 µm in (B). (C–J) Images of vegetative somatic cells from untagged control strain *Eve::VcMID* (C–F) or *Eve::VcMID-BH* (G–J), visualized by DIC light microscopy (C, G), stained with DAPI to visualize DNA in blue (D, H) or subject to IF with anti-HA in green (E, I). (F and J) are merged images of (D, E) and (H, I), respectively. The arrow in (H) shows the nucleus, while the smaller DAPI-stained regions are chloroplast DNA. The arrowhead in (I) shows the cytoplasmic VcMid signal that is excluded from the nucleus of the somatic cell. Scale bars = 7.5 µm.
(TIF)

Figure S10 Diagrams of *VcMID* hairpin constructs. (A) and (B) Sequences used to generate hp1 (A) and hp2 (B) from *VcMID* genomic and cDNA sequences. Exons E1 through E4 (blue filled boxes) and introns I1 through I4 (orange lines) were amplified and ligated together in the indicated orientations. (C) Diagram of *nitA* gene in pVcNR15 [71] with exons (E1–E11) indicated by thick black lines and intron 1 or UTR sequences as thin black lines. The Nde I site located in the 3′ UTR region was used as an insertion site for hairpin-forming sequences. (D) and (E) Diagrams of pVcMID-hp1 (D) and pVcMID-hp2 (E) derived from pVcNR15 showing inserted hairpin sequences from (A) and (B) in blue/orange.
(TIF)

Table S1 Zygote germination and viability in female × male and pseudo-female × male crosses.
(DOCX)

Table S2 Gamete differentiation in wild-type and transgenic female *Volvox* strains expressing Volvox and Chlamydomonas Mid proteins.
(DOCX)

Table S3 *Volvox* strains used in this study.
(DOCX)

Table S4 Oligonucleotides used in this study.
(DOCX)

Text S1 Supporting methods and references.
(DOCX)

Acknowledgments

We thank Takashi Hamaji for helpful discussion and reading of the manuscript. We thank Tuya Wulan, Fuqin Sun, and Richard Davenport for technical assistance.

Author Contributions

The author(s) have made the following declarations about their contributions: Conceived and designed the experiments: JGU SG PDH. Performed the experiments: SG PDH. Analyzed the data: JGU SG PDH. Contributed to the writing of the manuscript: JGU SG.

References

1. Hoekstra RF (1987) The evolution of sexes. Experientia Suppl 55: 59–91.
2. Parker GA (2011) The origin and maintenance of two sexes (anisogamy), and their gamete sizes by gamete competition. Togashi T, Cox PA, editors. The Evolution of Anisogamy. Cambridge University Press.
3. Ni M, Feretzaki M, Sun S, Wang X, Heitman J (2011) Sex in fungi. Annu Rev Genet 45: 405–430.
4. Umen JG (2011) Evolution of sex and mating loci: an expanded view from Volvocine algae. Curr Opin Microbiol 14: 634–641.
5. Cervantes MD, Hamilton EP, Xiong J, Lawson MJ, Yuan D, et al. (2013) Selecting one of several mating types through gene segment joining and deletion in *Tetrahymena thermophila*. PLoS Biol 11: e1001518.
6. Bloomfield G, Skelton J, Ivens A, Tanaka Y, Kay RR (2010) Sex determination in the social amoeba *Dictyostelium discoideum*. Science 330: 1533–1536.
7. Parker GA, Baker RR, Smith VG (1972) The origin and evolution of gamete dimorphism and the male-female phenomenon. Journal of Theoretical Biology 36: 529–553.
8. Randerson JP, Hurst LD (2001) The uncertain evolution of the sexes. Trends Ecol Evol 16: 571–579.
9. Billiard S, López-Villavicencio M, Devier B, Hood ME, Fairhead C, et al. (2011) Having sex, yes, but with whom? Inferences from fungi on the evolution of anisogamy and mating types. Biological reviews of the Cambridge Philosophical Society 86: 421–442.
10. Blute M (2012) The evolution of anisogamy: more questions than answers. Biol Theory 7: 3–9.
11. Charlesworth B (1978) The population genetics of anisogamy. J Theor Biol 73: 347–357.
12. Kirk DL (2005) A twelve-step program for evolving multicellularity and a division of labor. BioEssays 27: 299–310.
13. Bell G (1985) The origin and early evolution of germ cells as illustrated by the Volvocales. Halvorson HO, Monroy A, editors. The origin and evolution of sex. New York: Alan R. Liss Inc. pp. 221–256.
14. Goodenough U, Lin H, Lee J-H (2007) Sex determination in *Chlamydomonas*. Semin Cell Dev Biol 18: 350–361.
15. Nozaki H (1996) Morphology and evolution of sexual reproduction in the Volvocaceae (Chlorophyta). J Plant Res 109: 353–361.
16. Coleman AW (2012) A comparative analysis of the Volvocaceae (Chlorophyta). J Phycol 48: 491–513.
17. Knowlton N (1974) A note on the evolution of gamete dimorphism. J Theor Biol 46: 283–285.
18. Madsen JD, Waller DM (1983) A note on the evolution of gamete dimorphism in algae. American Naturalist 121: 443–447.
19. Wiese L, Wiese W, Edwards DA (1979) Inducible anisogamy and the evolution of oogamy from isogamy. Ann Bot 44: 131–139.
20. Ferris P, Olson BJSC, De Hoff PL, Douglass S, Casero D, et al. (2010) Evolution of an expanded sex-determining locus in *Volvox*. Science 328: 351–354.
21. Nozaki H, Mori T, Misumi O, Matsunaga S, Kuroiwa T (2006) Males evolved from the dominant isogametic mating type. Curr Biol 16: R1018–R1020.
22. Ning J, Otto TD, Pfander C, Schwach F, Brochet M, et al. (2013) Comparative genomics in *Chlamydomonas* and *Plasmodium* identifies an ancient nuclear envelope protein family essential for sexual reproduction in protists, fungi, plants, and vertebrates. Genes Dev 27: 1198–1215.
23. Ferris PJ, Goodenough UW (1994) The mating-type locus of *Chlamydomonas reinhardtii* contains highly rearranged DNA sequences. Cell 76: 1135–1145.
24. De Hoff PL, Ferris P, Olson BJSC, Miyagi A, Geng S, et al. (2013) Species and population level molecular profiling reveals cryptic recombination and emergent asymmetry in the dimorphic mating locus of *C. reinhardtii*. PLoS Genet 9: e1003724.
25. Ferris PJ, Goodenough UW (1997) Mating type in *Chlamydomonas* is specified by mid, the minus-dominance gene. Genetics 146: 859–869.
26. Lin H, Goodenough UW (2007) Gametogenesis in the *Chlamydomonas reinhardtii* minus mating type is controlled by two genes, MID and MTD1. Genetics 176: 913–925.
27. Ferris PJ, Pavlovic C, Fabry S, Goodenough UW (1997) Rapid evolution of sex-related genes in *Chlamydomonas*. Proc Natl Acad Sci U S A 94: 8634–8639.
28. Hamaji T, Ferris PJ, Coleman AW, Waffenschmidt S, Takahashi F, et al. (2008) Identification of the minus-dominance gene ortholog in the mating-type locus of *Gonium pectorale*. Genetics 178: 283–294.
29. Setohigashi Y, Hamaji T, Hayama M, Matsuzaki R, Nozaki H (2011) Uniparental inheritance of chloroplast DNA is strict in the isogamous volvocalean *Gonium*. PLoS ONE 6: e19545.
30. Hamaji T, Ferris PJ, Nishii I, Nishimura Y, Nozaki H (2013) Distribution of the sex-determining gene MID and molecular correspondence of mating types within the isogamous genus *Gonium* (Volvocales, Chlorophyta). PLoS ONE 8: e64385.
31. Kirk DL, Nishii I (2001) *Volvox carteri* as a model for studying the genetic and cytological control of morphogenesis. Dev Growth Differ 43: 621–631.
32. Kirk DL (2001) Germ–soma differentiation in *Volvox*. Dev Biol 238: 213–223.
33. Kochert G, Yates I (1974) Purification and partial characterization of a glycoprotein sexual inducer from *Volvox carteri*. Proc Natl Acad Sci U S A.
34. Starr RC, Jaenicke L (1974) Purification and characterization of the hormone initiating sexual morphogenesis in *Volvox carteri* f. *nagariensis* Iyengar. Proc Natl Acad Sci U S A 71: 1050–1054.
35. Mages HW, Tschochner H, Sumper M (1988) The sexual inducer of *Volvox carteri*. Primary structure deduced from cDNA sequence. FEBS Letters 234: 407–410.
36. Starr RC (1969) Structure, reproduction and differentiation in Volvox carteri f. nagariensis Iyengar, strains HK9 & 10. Arch Protistenkd 111: 204–222.
37. Kochert G (1968) Differentiation of reproductive cells in *Volvox carteri*. J Protozool 15: 438–452.
38. Starr RC (1975) Meiosis in *Volvox carteri* f. *nagariensis*. Archiv fur Protistenkunde 117: 187–191.
39. Bachtrog D, Kirkpatrick M, Mank JE, McDaniel SF, Pires JC, et al. (2011) Are all sex chromosomes created equal? Trends Genet 27: 350–357.
40. Hiraide R, Kawai-Toyooka H, Hamaji T, Matsuzaki R, Kawafune K, et al. (2013) The evolution of male-female sexual dimorphism predates the gender-based divergence of the mating locus gene MAT3/RB. Mol Biol Evol 30: 1038–1040.
41. Starr RC, Jaenicke L (1989) Cell differentiation in *Volvox carteri* (Chlorophyceae): the use of mutants in understanding patterns and control. Algae as experimental systems. New York: Alan R. Liss Inc. pp. 135–147.
42. Nishii I, Miller SM (2010) *Volvox*: simple steps to developmental complexity? Curr Opin Plant Biol 13: 646–653.
43. Umen JG, Olson BJSC (2012) Genomics of volvocine algae. Piganeau G, editor. Genomic insights into the biology of algae. New York: Elsevier, Vol. 64. pp. 185–243.
44. Shubin N, Tabin C, Carroll S (2009) Deep homology and the origins of evolutionary novelty. Nature 457: 818–823.
45. Gehring WJ (2005) New perspectives on eye development and the evolution of eyes and photoreceptors. J Hered 96: 171–184.
46. Herron MD, Hackett JD, Aylward FO, Michod RE (2009) Triassic origin and early radiation of multicellular volvocine algae. Proc Natl Acad Sci U S A 106: 3254–3258.
47. McCourt RM, Delwiche CF, Karol KG (2004) Charophyte algae and land plant origins. Trends Ecol Evol (Amst) 19: 661–666.
48. Levin TC, King N (2013) Evidence for sex and recombination in the choanoflagellate *Salpingoeca rosetta*. Curr Biol 23: 2176–2180.
49. Nakada T, Shinkawa H, Ito T, Tomita M (2009) Recharacterization of *Chlamydomonas reinhardtii* and its relatives with new isolates from Japan. J Plant Res 123: 67–78.
50. Pires ND, Dolan L (2012) Morphological evolution in land plants: new designs with old genes. Philos Trans R Soc Lond B Biol Sci 367: 508–518.
51. Davidson EH (2006) Gene regulatory networks and the evolution of animal body plans. Science 311: 796–800.
52. Wray GA (2003) Transcriptional regulation and the evolution of development. Int J Dev Biol 47: 675–684.
53. Monteiro A (2012) Gene regulatory networks reused to build novel traits. BioEssays 34: 181–186.
54. Aravind L, Anantharaman V, Venancio TM (2009) Apprehending multicellularity: regulatory networks, genomics, and evolution. Birth Defects Res C Embryo Today 87: 143–164.
55. Starr R (1968) Cellular differentiation in *Volvox*. Proc Natl Acad Sci U S A 59: 1082–1088.
56. Schauser L, Wieloch W, Stougaard J (2005) Evolution of NIN-like proteins in *Arabidopsis*, rice, and *Lotus japonicus*. J Mol Evol 60: 229–237.
57. Worden AZ, Lee J-H, Mock T, Rouzé P, Simmons MP, et al. (2009) Green evolution and dynamic adaptations revealed by genomes of the marine picoeukaryotes *Micromonas*. Science 324: 268–272.
58. Curtis BA, Tanifuji G, Burki F, Gruber A, Irimia M, et al. (2012) Algal genomes reveal evolutionary mosaicism and the fate of nucleomorphs. Nature 492: 59–65.
59. Smith GM (1944) A comparative study of the species of *Volvox*. Transactions of the American Microscopical Society 63: 265–310.
60. Campbell AM, Rayala HJ, Goodenough UW (1995) The iso1 gene of *Chlamydomonas* is involved in sex determination. Mol Biol Cell 6: 87–95.
61. Ferris PJ, Woessner JP, Goodenough UW (1996) A sex recognition glycoprotein is encoded by the plus mating-type gene fus1 of *Chlamydomonas reinhardtii*. Mol Biol Cell 7: 1235–1248.
62. Nozaki H, Ott FD, Coleman AW (2006) Morphology, molecular phylogeny and taxonomy of two new species of Pleodorina (Volvoceae, Chlorophyceae). J Phycol 42: 1072–1080.
63. Al-Hasani H, Jaenicke L (1992) Characterization of the sex-inducer glycoprotein of *Volvox carteri* f. *weismannia*. Sex Plant Reprod 5: 8–12.
64. Bull J (1978) Sex chromosomes in haploid dioecy: a unique contrast to Muller's theory for diploid dioecy. Am Nat 112: 245–250.
65. Rice W (1984) Sex chromosomes and the evolution of sexual dimorphism. Evolution 38: 735–742.
66. Yamato KT, Ishizaki K, Fujisawa M, Okada S, Nakayama S, et al. (2007) Gene organization of the liverwort Y chromosome reveals distinct sex chromosome evolution in a haploid system. Proc Natl Acad Sci U S A 104: 6472–6477.

67. Prochnik SE, Umen J, Nedelcu AM, Hallmann A, Miller SM, et al. (2010) Genomic analysis of organismal complexity in the multicellular green alga *Volvox carteri*. Science 329: 223–226.
68. Umen JG, Goodenough UW (2001) Control of cell division by a retinoblastoma protein homolog in *Chlamydomonas*. Genes Dev 15: 1652–1661.
69. Fang S-C, de los Reyes C, Umen JG (2006) Cell size checkpoint control by the retinoblastoma tumor suppressor pathway. PLoS Genet 2: e167.
70. Sambrook JJ, Russell DW (2001) Molecular cloning: a laboratory manual. 3rd edition. Cold Spring Harbor (New York): CSHL Press. 1 pp.
71. Gruber H, Kirzinger S, Schmitt R (1996) Expression of the *Volvox* gene encoding nitrate reductase: Mutation-dependent activation of cryptic splice sites and intron-enhanced gene expression from a cDNA. Plant Mol Biol 31: 1–12.
72. Schiedlmeier B, Schmitt R, Müller W, Kirk MM, Gruber H, et al. (1994) Nuclear transformation of *Volvox carteri*. Proc Natl Acad Sci U S A 91: 5080–5084.
73. Huskey RJ, Griffin BE, Cecil PO, Callahan AM (1979) A preliminary genetic investigation of *Volvox carteri*. Genetics 91: 229–244.
74. Edgar RC (2004) MUSCLE: a multiple sequence alignment method with reduced time and space complexity. BMC Bioinformatics 5: 113.
